# Impact of Ribosomal Modification on the Binding of the Antibiotic Telithromycin Using a Combined Grand Canonical Monte Carlo/Molecular Dynamics Simulation Approach

**DOI:** 10.1371/journal.pcbi.1003113

**Published:** 2013-06-13

**Authors:** Meagan C. Small, Pedro Lopes, Rodrigo B. Andrade, Alexander D. MacKerell

**Affiliations:** 1 Department of Pharmaceutical Sciences, University of Maryland, Baltimore, Maryland, United States of America; 2 Department of Chemistry, Temple University, Philadelphia, Pennsylvania, United States of America; CNAG - Centre Nacional d'Anàlisi Genòmica and CRG - Centre de Regulació Genòmica, Spain

## Abstract

Resistance to macrolide antibiotics is conferred by mutation of A2058 to G or methylation by Erm methyltransferases of the exocyclic N6 of A2058 (E. coli numbering) that forms the macrolide binding site in the 50S subunit of the ribosome. Ketolides such as telithromycin mitigate A2058G resistance yet remain susceptible to Erm-based resistance. Molecular details associated with macrolide resistance due to the A2058G mutation and methylation at N6 of A2058 by Erm methyltransferases were investigated using empirical force field-based simulations. To address the buried nature of the macrolide binding site, the number of waters within the pocket was allowed to fluctuate via the use of a Grand Canonical Monte Carlo (GCMC) methodology. The GCMC water insertion/deletion steps were alternated with Molecular Dynamics (MD) simulations to allow for relaxation of the entire system. From this GCMC/MD approach information on the interactions between telithromycin and the 50S ribosome was obtained. In the wild-type (WT) ribosome, the 2′-OH to A2058 N1 hydrogen bond samples short distances with a higher probability, while the effectiveness of telithromycin against the A2058G mutation is explained by a rearrangement of the hydrogen bonding pattern of the 2′-OH to 2058 that maintains the overall antibiotic-ribosome interactions. In both the WT and A2058G mutation there is significant flexibility in telithromycin's imidazole-pyridine side chain (ARM), indicating that entropic effects contribute to the binding affinity. Methylated ribosomes show lower sampling of short 2′-OH to 2058 distances and also demonstrate enhanced G2057-A2058 stacking leading to disrupted A752-U2609 Watson-Crick (WC) interactions as well as hydrogen bonding between telithromycin's ARM and U2609. This information will be of utility in the rational design of novel macrolide analogs with improved activity against methylated A2058 ribosomes.

## Author Summary

Bacterial resistance to antibiotics is a serious public health problem that requires the continuous development of new antibiotics. Bacteria acquire resistance to macrolide antibiotics by (1) effluxing the drug from the cell, (2) modifying the drug, or (3) modifying the drug target (i.e., the 50S subunit of the ribosome) to abrogate or completely abolish binding. While newer antibiotics are able to avoid the first two mechanisms, they remain unable to overcome resistance due to ribosomal modification, particularly due to methyltransferase (i.e., *erm*) enzymes. We have applied computer-aided drug design methods designed explicitly for studies of the ribosome to better understand the relationship between modification of the ribosome by *erms* and the binding of telithromycin, a 3^rd^ generation ketolide antibiotic derived from erythromycin. While we confirm that ribosomal modification leads to decreased binding due to disruption of key interactions with the drug, we find these modifications effect a structural rearrangement of the entire region of the ribosome responsible for binding macrolide antibiotics. This information will be useful in the design of novel antibiotics that are effective against resistant bacteria possessing modified ribosomes.

## Introduction

Microbial resistance presents a major challenge in the development of novel antibiotics because bacteria are continually developing new resistance mechanisms [1]. The bacterial ribosome is a target for over 60% of antibiotics [2], [3], which bind at vital sites within both the 30S and 50S ribosomal subunits and inhibit processes that are essential for cell survival [4]–[10]. One important class of antibiotics is the macrolides, which bind at the beginning of the exit tunnel in the 50S subunit of the bacterial ribosome and block elongation of the nascent polypeptide chain [10]–[12]. For the macrolide class of antibiotics, bacteria achieve resistance by modifying or mutating bases within the binding pocket as well as by other mechanisms such as drug metabolism and overexpression of efflux pumps. Telithromycin is the first of a new class of macrolides, called the ketolides, named for the substitution of the C3 L-cladinose sugar with a ketone. Ketolides have largely addressed resistance due to drug efflux and metabolism yet still remain susceptible to ribosomal modification [13], [14], the most clinically relevant of which is modification of A2058 in 23S rRNA (*E. coli* numbering throughout) that confers cross-resistance to antibiotics in the macrolide, lincosamide and streptogramin B classes (MLS) [15]–[18]. A2058-based resistance includes mutation of A2058 to G as well as methylation of the exocyclic N6 of A2058 via the expression of *erm* genes encoding methyltransferases (Erm methyltransferases) that add one or two methyl groups and represent the most effective mechanism of macrolide resistance [19]–[21]. Telithromycin and other ketolides have been found to mitigate macrolide resistance due to the A2058G mutation (**Table S1 in Text S1**) [22]–[24], though ketolides that are widely effective against Erm methylation-based modifications are not known. Still, telithromycin has shown improved activity against monomethylated ribosomes compared to previous generation macrolides (**Table S1 in Text S1**) [23], [25], which has been attributed to secondary contacts made between telithromycin's alkyl-aryl group and bases A752 and U2609 within the binding pocket [15], [26]–[28].

Telithromycin's improved activity compared to erythromycin can also partly be explained by its absence of *erm* gene induction [29]. Erythromycin has been shown to increase levels of Erm methyltransferases in species carrying inducible *erm* genes [17], [18], [25], [30]–[34] consequently increasing the levels of dimethylated ribosomes, whereas telithromycin and other ketolides have been found to mostly bypass the *erm* gene induction pathway [29], [34], [35]. In a comprehensive study looking at antibiotic activity as a function of mono-/dimethylated 2058 ribosome levels, it was found that in general increased monomethyl A2058 levels alone were not sufficient to mitigate telithromycin activity and that upon increasing the percentage of dimethylated ribosomes via induction with erythromycin was significant telithromycin susceptibility observed [25]. However, species-dependent exceptions were noted and therefore it appears that a combination of factors is involved in enhancing telithromycin's effectiveness in monomethylated ribosomes. Even so, dimethylated ribosomes exhibit the most potent resistance to telithromycin [25] as well as other antibiotics in the MLS class [18] and higher levels of dimethylated ribosomes confer the greatest resistance. Given the widespread transfer of resistant genes between species [36], [37] it is important to develop analogs with improved affinity toward Erm-mediated resistant phenotypes.

**Figure 1:**
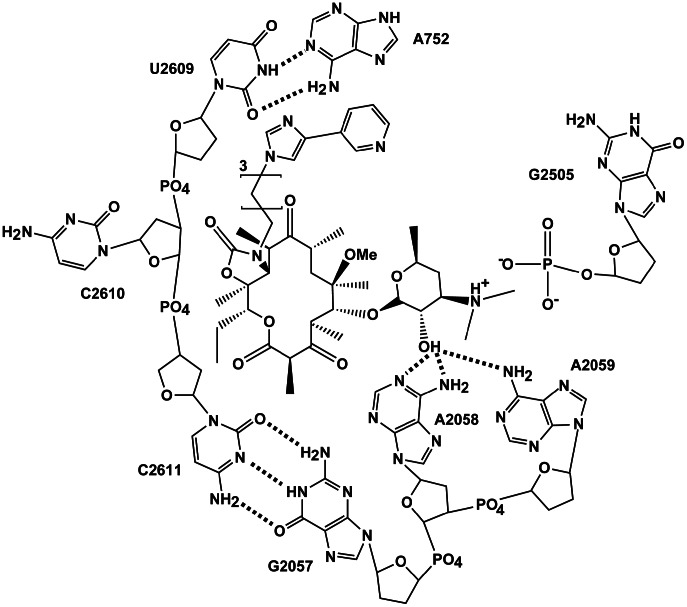
2D representation of telithromycin and bases within the macrolide binding pocket showing the three biologically relevant telithromycin-ribosome interactions that are the subject of this study: 2′-OH to 2058/2059 hydrogen bonding, stacking between telithromycin's ARM and A752-U2609 WC base pair, and ionic interactions between telithromycin's 3′-protonated dimethylamine and G2505. Dotted lines represent hydrogen bonding. Hydroxyl groups of the ribose sugars are omitted for clarity.

Crystal structures of telithromycin bound to *E. coli* [38] (as well as *T. thermophilus* [39], *H. marismortui* [15], and *D. radiodurans* [10]) have revealed several interactions that are important for telithromycin binding, as shown schematically in **Figure 1**. Multiple van der Waals (VDW) contacts between hydrophobic alkyl groups on the macrolactone and residues forming the walls of the exit tunnel are present that contribute to binding. Telithromycin's desosamine 2′-hydroxyl group forms hydrogen bonds with N1 of A2058, N6 of A2058, and N6 of neighboring A2059 [10]. In addition, desosamine's 3′-dimethylamino moiety forms a salt bridge with the phosphate of G2505 [10]. And, in the E. coli [38] crystal structure the imidazole-pyridine side-chain (ARM) extending from the C11–C12 cyclic carbamate engages in stacking with A752 and U2609, increasing telithromycin's binding affinity compared to earlier generation macrolides such as clarithromycin, roxithromycin, and azithromycin [15], [26]–[28]. The present study addresses the molecular details associated with macrolide resistance due to the A2058G mutation and methylation at N6 of A2058 by Erm methyltransferases. To compare these interactions in the wild type and telithromycin-resistant strains, empirical force field-based simulations were performed on truncated versions of the wild type and mutant/modified *E. coli* 50S ribosomal subunits. The E. coli crystal structure [38] is selected because it is the first crystal structure available for a pathogenic bacterial species and hence most relevant in terms of drug design. To address the buried nature of the macrolide binding site, the number of waters within the pocket were allowed to fluctuate via the use of a combined Grand Canonical Monte Carlo (GCMC)/Molecular Dynamics (MD) methodology that builds on previous methodological developments [40]–[50]. GCMC/MD has been successfully applied to systems with deeply buried binding pockets [51], [52] and has been applied to the ribosome to determine the binding free energy of sparsomycin [53], [54]. It is utilized here as a means to assure proper solvation of the telithromycin binding site. The GCMC water insertion/deletion steps are alternated with MD simulations to allow for relaxation of the entire system. From this GCMC/MD approach information on the nature of the interactions between telithromycin and the 50S ribosome was obtained, yielding a detailed understanding of these therapeutically relevant antibiotic resistance mechanisms at the molecular level.

## Results/Discussion

The present work sets out to understand details of the impact of ribosome modifications on the ketolide antibiotic telithromycin using MD simulations. For computational expediency, as required to obtain adequate conformational sampling, the ribosomal system was truncated to a 40 Å radius spherical system centered on telithromycin and a GCMC/MD approach was used to assure proper solvation of the system. Validation of the methodology along with tests of convergence of the simulations are detailed in the Supporting Information (Text S1). Applying this methodology allowed for 150 ns of MD sampling of each of the studied species, with analysis focusing on the interactions between telithromycin and the ribosome shown in **Figure 1** and how those interactions impact the local structure of the ribosome, thereby leading to altered telithromycin-ribosome binding modes.

We note that ideally the relative free energies of binding of telithromycin to the studied forms of the ribosome would be calculated, including the energetic impact of solvation on the overall binding affinity. However, determining free energies in a complex system such as the ribosome is difficult. For example, in the present study we investigate the impact of ribosomal mutation or methylation on binding rather than changes in the ligand (ie. telithromycin). Thus, the contribution of differential desolvation penalties to the overall activity is associated with chemical changes in the A2058 base in the ribosome environment. To obtain estimates of the changes in solvation it would be necessary to perform free energy perturbations in the context of a GCMC/MD formalism between WT, A2058G and methylated bases in the ribosome in the absence of antibiotic. This is a very challenging calculation, which would be further complicated by the likely presence of various ions in the absence of telithromycin that would impact the solvation energy. These and other considerations led us to focus our analysis on structural/interaction contributions to binding rather than quantitative estimates of changes in the free energy of binding.

**Figure 2:**
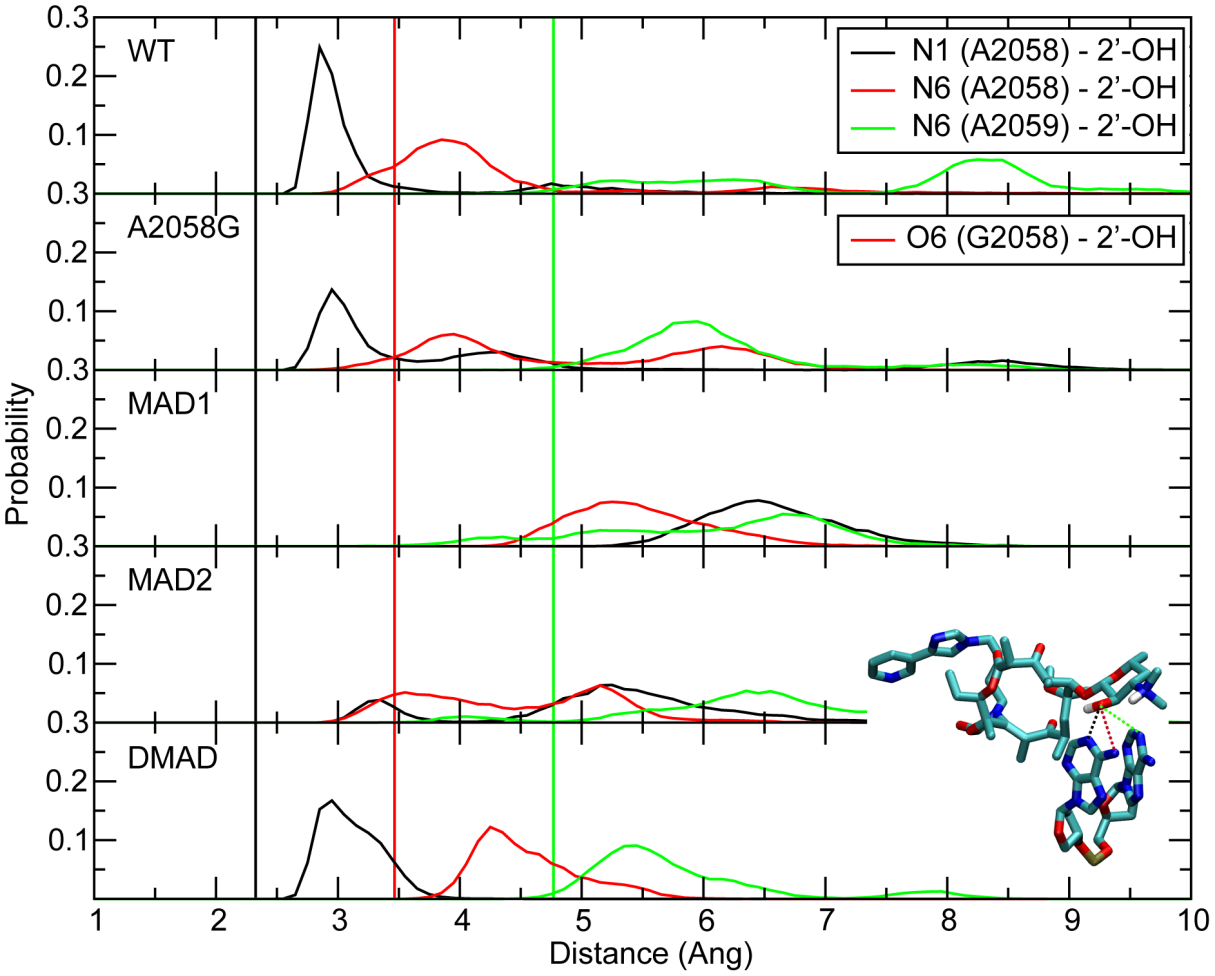
The probability distributions of the telithromycin 2′OH to A2058 N1, A2058 N6 and A2059 N6 distances for WT, A2058G mutant and methyl modifications. The 2′-OH – A2058 N1 distance is shown in black, 2′-OH to A2058 N6/O6 in red, and 2′-OH to A2059 N6 in green. Distances reported are measured between heavy atoms. Corresponding crystal structure distances are shown as vertical lines using the same color scheme.

Initial analysis involved the hydrogen bonds between telithromycin's desosamine hydroxyl (2′-OH) and A2058 N1/N6 or A2059 N6 as these have been indicated to play a vital role in telithromycin binding and are presumably disrupted in A2058-modified ribosomes [15], [38], [39]. Probability distributions of the 2′-OH to N distances are shown in **Figure 2** for the WT and A2058 mutated/modified bacterial ribosomes. Distances for the WT simulation are in good agreement with the crystal structure distances. The maximum in the 2′-OH to A2058 N1 probability distribution occurs at 2.95 Å versus the crystal structure distance of 2.32 Å. The crystal distance, which is short for a hydrogen bond (i.e. WC N1-N3 interaction distances are approximately 2.9 Å [55]) is probably explained by the resolution of the crystal structure (3.25 Å) obtained at a temperature of 100 K. In addition, the average B factors for telithromycin and A2058 (base atoms only) are 28.5 and 31.4, respectively, which correspond to root mean square fluctuations of nearly 1 Å each at the temperature of 100 K. The 2′-OH to A2058 N6 WT distance is also in good agreement with the crystal data. Notably the 2′-OH to N6 interaction is not direct, as indicted by the interaction distances being >3 Å. There is also an interaction between the 2′-OH and A2059 N6, which samples a range of distances in the simulation compared to a crystal structure interaction distance of ∼4.7 Å indicating that it is a relatively weak interaction. The 2′-OH to N6 A2059 hydrogen bond was proposed based on crystal structures of other macrolides (e.g., erythromycin, clarithromycin, and roxithromycin) bound to the *Deinococcus radiodurans* 50S subunit [10], but does not play a significant role here.

Telithromycin activity is slightly decreased in the A2058G mutant [23], [24]. Consistent with this is the distance probability distributions in **Figure 2** showing the hydrogen bonding to be largely maintained. This is somewhat surprising given that upon going from A to G the N1 atom becomes a donor and the exocyclic N6 amine of A is replaced by a keto group in G. The maintenance of the 2′-OH to N1 interaction in A2058G is due to the 2′-hydroxyl now acting as an acceptor and the corresponding reorientation of the hydroxyl proton allowing it to form a favorable hydrogen bond with the O6 of G. Thus, the 2′-hydroxyl in telithromycin can accommodate the switch in the location of the donor and acceptor groups in the A2058G mutation thereby maintaining favorable hydrogen bonding and explaining how telithromycin remains effective against A2058G mutants.

Erm-dependent methylation of A2058 leads to telithromycin resistance, with activity diminished in monomethylated ribosomes and almost completely abolished in dimethylated ribosomes [23]. For monomethylated ribosomes, the N6-methyl group of A2058 can be oriented toward the desosamine sugar or away from it and QM calculations (**Table S5 in Text S1**) indicate both orientations to be energetically accessible, though the barrier between the orientations is high. Accordingly, both orientations of the N6 monomethyl modifications were explicitly studied, where the methyl group is directed either toward (MAD1) or away from (MAD2) the desosamine sugar (i.e., cis or trans to A2058 N1, respectively). The hydrogen bonding distributions for the methylated species are included in **Figure 2**. With the N6 monomethylations (i.e., MAD1 and MAD2), the hydrogen bonding is significantly perturbed, with that perturbation larger in MAD1, consistent with the methyl being directed towards telithromycin. However, in the N,N′-dimethylated species the hydrogen bonding pattern is well maintained. The N1 to 2′-OH distribution is only slightly perturbed from that in the WT and the N6 to 2′-OH interaction is also well maintained, with a shift to longer distances as compared to the WT due to the presence of the methyls. Thus, while the perturbation of the A2058-telithromycin interactions in the monomethyl modifications is consistent with their lower activity, the maintenance of interactions with the N,N′-dimethyl modification indicate additional factors are impacting the efficacy of telithromycin against the methylated species.

**Figure 3:**
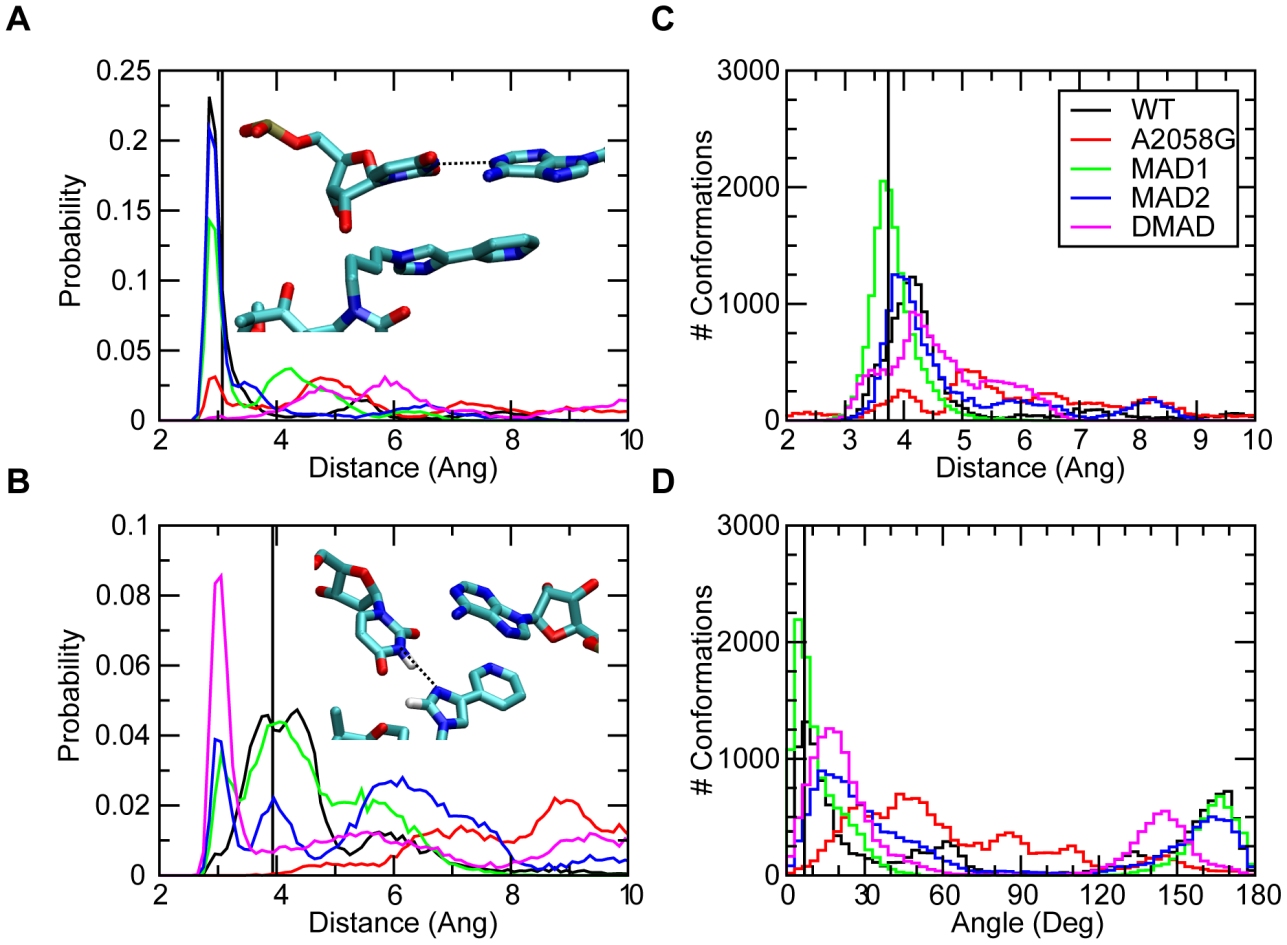
The probability distributions for WT, A2058G mutant and methyl modifications of the (A) WC A752 N1 to U2609 N3 distances, (B) telithromycin imidazole to U2609 N3 distances, (C) distance between the centers of masses of A752-U2609 and telithromycin's imidazole-pyridine (ARM) moiety and (D) angle between the planes defined A752-U2609 and the imidazole-pyridine moieties. The crystal structure values are shown as a vertical black line, while the distributions from the GCMC/MD simulations are shown as WT (black), A2058G (red), MAD1 (green), MAD2 (blue), and DMAD (magenta).

With this in mind, the interactions of telithromycin's imidazole-pyridine moiety (ARM) with A752-U2609 were investigated. Telithromycin's increased affinity for the 50S ribosome macrolide binding pocket compared to earlier generation macrolides has been linked to these secondary interactions [15], [38], [39]. Crystal structures show that the heterocyclic ARM stacks with U2609 and A752 [38], [39], (**Figure 1**), which themselves form a Watson-Crick (WC) pair that bridge domains II and IV of 23S rRNA [38]. Accordingly, the properties of A752-U2609 and the ARM were compared between the WT and mutant/modified ribosomes. The A752 N1 to U2609 N3 WC distance (**Figure 3a**) is well maintained in both the WT and MAD2 systems, though some sampling of longer distances is evident. A2058G and the other methylated species undergo a significant loss of WC base pairing, with MAD1 followed by A2058G still sampling some of the WC state. Concerning A752-U2609 to ARM interactions, in the simulations stacking was observed to varying degrees in the different systems. Probability distributions of the COM distances and angles between least-square planes through the A752-U2609 and the ARM (**Figure 3c,d**) show a significant amount of stacking in all the systems except A2058G. However, in all the systems a wide range of spatial arrangements of the bases and the ARM beyond the stacked state are being sampled. This is associated with high fluctuations of the ARM, as evidenced by the RMS fluctuations of telithromycin in the different systems (**Figure S9 in Text S1**). The highest fluctuations occur with the WT and A2058G systems, with the magnitude of the fluctuations consistent with those obtained from the crystallographic study based on the B factors. Notably, the fluctuations of the ARM, which were obtained at 100 K in the experimental study, are larger than the remainder of the ketolide, indicating that the ARM is likely sampling a range of conformations, including the stacked state, as observed in the simulations. Interestingly, the methylations have the lowest fluctuations of the ARM. Further analysis of A752-U2609 to ARM interactions revealed the presence of hydrogen bonds between U2609 and the imidazole ring of the ARM (**Figure 3b**). Thus, the methylations lead to perturbations of the region of the ribosome around the ARM that lead to ARM-U2609 hydrogen bonding, thereby contributing to the lowered fluctuations of the ARM, while the WT and A2058G systems have a highly flexible ARM. Given the activity of telithromycin against these species, these results indicate that the flexibility is important for activity. However, the presence of the hydrogen bonds between the ARM and U2609 in the methylated species has interesting implications in the context of ligand design, as discussed below.

**Figure 4:**
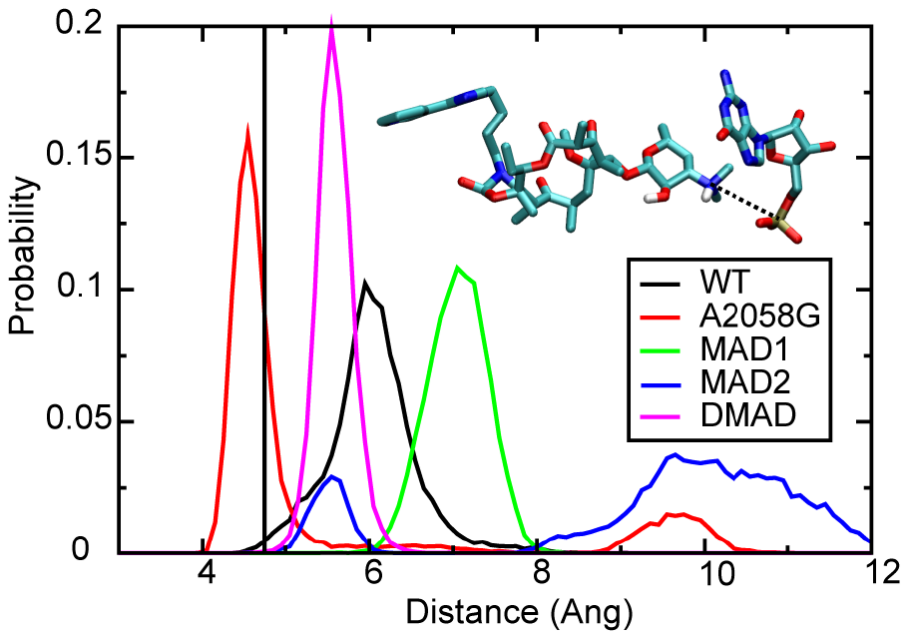
The probability distributions of distances between telithromycin's 3′-protonated dimethylamine and G2505 P for WT, A2058G mutant and methyl modifications. The crystal structure distance is shown as a vertical black line, while the distributions are from the GCMC/MD simulations: WT (black), A2058G (red), MAD1 (green), MAD2 (blue), and DMAD (magenta).

The third interaction considered is the electrostatic interaction between telithromycin's 3′-protonated dimethylamine and the phosphate of G2505 (**Figure 1**) [10]. Distance distributions for the 3′-nitrogen to the phosphate are shown in **Figure 4**. WT and MAD1 sample the shortest distances (∼3.5 Å) with A2058G and DMAD also forming stable interactions. While WT and MAD1 do sample shorter distances than the other species, it is not evident that this interaction discriminates between the WT, A2058G, MAD1 and DMAD species. The favorable interactions of MAD1 may contribute to telithromycin maintaining weak activity against the monomethylated species [25], while the systematically longer distances with MAD2 may contribute to a loss of binding of telithromycin to that species.

**Figure 5:**
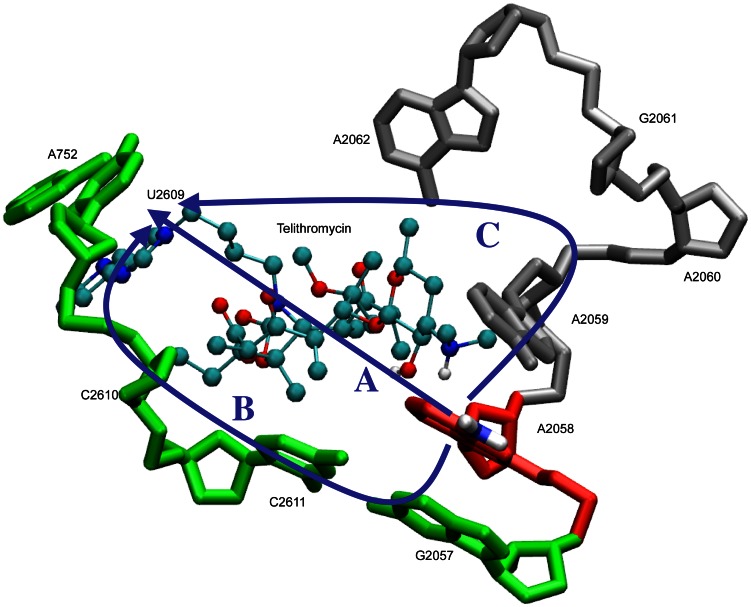
Proposed communication pathways from A2058 to the A752-U2609 and ARM region: the telithromycin pathway (A), G2057 pathway (B), or A2059 pathway (C).

While the hydrogen bonding of 2058 with telithromycin and the ionic interaction involving G2505 are direct interactions between the ribosome and the antibiotic that may be directly perturbed by mutation or methylation, the significant changes in the A752-U2609 region were somewhat unexpected. Accordingly, further analysis was performed to understand how alteration of A2058 leads to long-range changes in telithromycin-ribosome interactions. Inspection of the telithromycin-ribosome structure suggested three potential “communication” pathways, as shown in **Figure 5**. The first, the telithromycin pathway, involves communication through telithromycin. The second pathway, or G2057 pathway, involves A2058 stacking with G2057 that may be communicated to A752-U2609 via the covalent connectivity from C2611 through U2609. And the third pathway, or A2059 pathway, proceeds via the covalent connectivity from A2058 through A2062 to telithromycin's ARM via VDW interactions. To determine possible contributions of these three possibilities, each pathway was subjected to further analysis.

**Figure 6:**
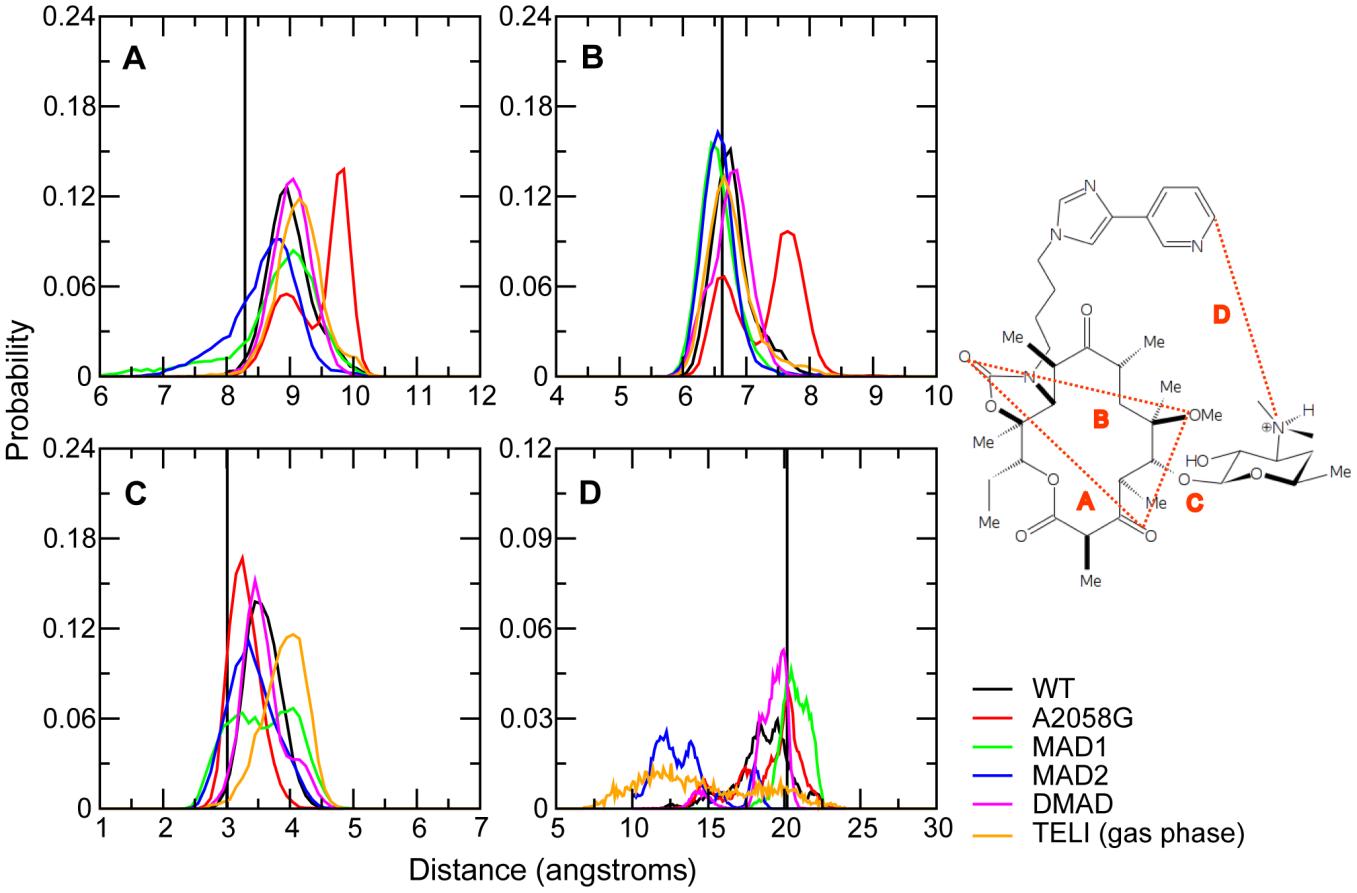
The probability distributions for WT, A2058G mutant and methyl modifications of telithromycin intramolecular distances (A) C3-O to C16-O, (B) C16-O to C6-O, (C) C3-O to C6-O, and (D) C3′-N and C26 as shown in the inset figure. The crystal structure values are shown as vertical black lines, while distributions are from the GCMC/MD simulations: WT (black), A2058G (red), MAD1 (green), MAD2 (blue), and DMAD (magenta). The probability distributions from telithromycin gas phase simulations without the ribosome are shown in orange.

Analysis of the telithromycin pathway involved inspection of the conformational sampling of the antibiotic in the simulations. Conformational sampling of the macrolactone ring was similar for the studied species, being consistent with that observed in the crystal structure (**Figure 6**), although a second conformation is observed in A2058G. Significantly, a much greater range of distances was sampled by the ARM, with those distances varying significantly from that in the crystal structure, consistent with the stacking and RMS fluctuation analysis discussed above. Given the similarities of the conformation of telithromycin in the different systems, the telithromycin pathway does not appear to be the primary communication pathway between the A2058 and the ARM regions, with a possible exception of the A2058G species.

**Figure 7:**
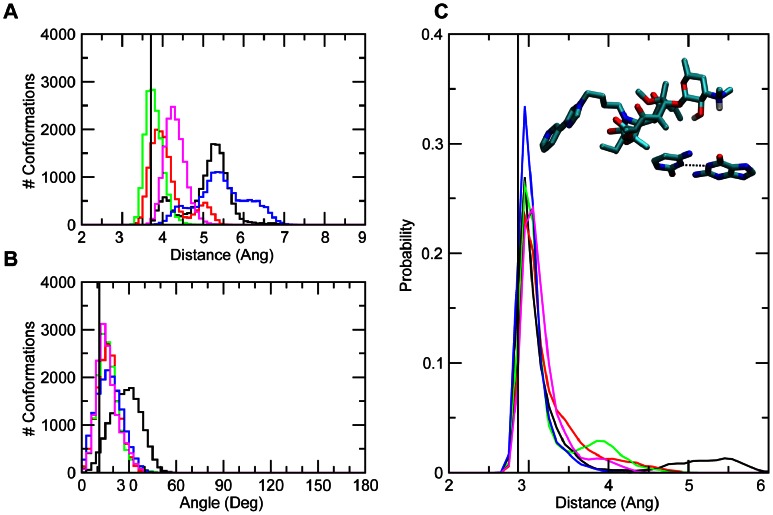
The number of conformations for WT, A2058G mutant and methyl modifications of (A) COM distances between the base atoms of 2057 and 2058 and (B) angles between planes comprised by 2057 and 2058 base atoms. The probability distributions for (C) WC G2057 N1 to C2611 N3 distances. The crystal structure values are shown as vertical black lines, while distributions from the GCMC/MD simulations are shown as WT (black), A2058G (red), MAD1 (green), MAD2 (blue), and DMAD (magenta).

Central to the G2057 (i.e., second pathway) is the stacking of A2058 with G2057 and the WC interaction between G2057 and C2611 (**Figure 1**). This offers a logical, through-bond path by which perturbations of A2058 may impact RNA-telithromycin ARM interactions. Analysis of the stacking interactions involved investigation of the center of mass (COM) distances and the angles between least square planes through the respective bases (**Figure 7a,b**). The probability distributions show that the WT samples a bimodal distance distribution with one mode corresponding to the stacked state observed in the crystal. In contrast, A2058G, MAD1 and DMAD sample single distributions in the vicinity of the crystal value, indicating improved stacking. A single distribution also occurs in MAD2 though a wide range of COM distances are sampled in that system. These results indicate increased stacking between A2058 and G2057 in A2058G, MAD1 and DMAD. Less favorable stacking with MAD2 is due to the N6 methyl directed towards the G2057 base, thereby perturbing stacking, while the second methyl in DMAD overcomes this leading to enhanced stacking. Thus, in all modified systems alteration of the interactions between the bases of A2058 and G2057 are present.

**Figure 8:**
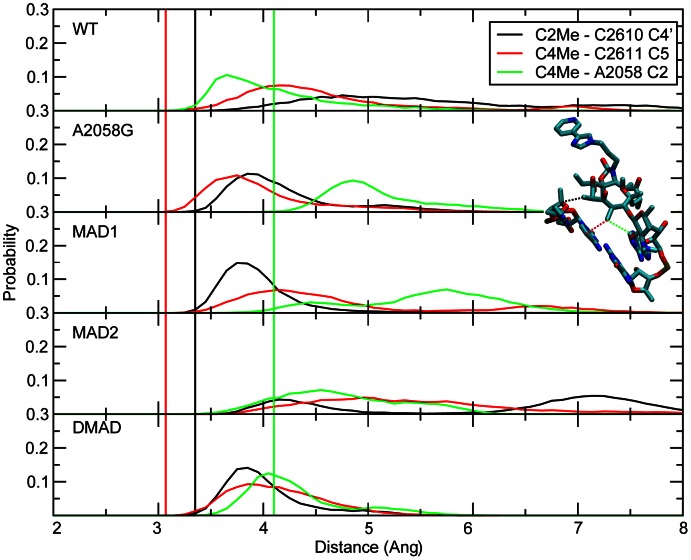
The probability distributions for WT, A2058G mutant and methyl modifications of the telithromycin C2-methyl to C2610 C4′ (black), C4-methyl to C2611 C5 (red), and C4-methyl to A2058 C2 (green). Corresponding crystal structure values are shown as vertical lines using the same color scheme.

Perturbation of A2058-G2057 interactions would be communicated along the G2057 pathway via WC interactions of that base with C2611. WC interactions between GC are high in all the systems indicating maintenance of base pairing (**Figure 7c**). This is consistent with experimental studies indicating that the orientation of G2057 and its base pairing with C2611 are important for maintaining the orientation of 2058, with the overall conformation of RNA being perturbed upon mutation of these bases [56], [57]. This allows the mutation/modifications to impact the orientation of C2611, C2610 and U2609. C2610 and C2611 make VDW contacts with two of telithromycin's methyl groups, C2 and C4 (**Figure 1**), which are 3.3 and 3.0 Å from the nearest non-hydrogen atoms on C2610 and C2611, respectively. In addition, C4 is also close to A2058, being 4.2 Å away in the crystal structure. Notably, in earlier generation macrolides the disruption of this interaction was thought to contribute to the loss of activity in A2058G mutants [16]. To investigate the impact of the A2058 mutation and methylations on these interactions, their probability distributions were calculated (**Figure 8**). In all the systems there is an outward shift in the two shortest interactions by ∼1 Å suggesting that the 100 K crystal structure distances are slightly too short, while in the WT the C4 to A2058 C2 interaction became shorter. In the A2058G mutant, the two short interactions are well maintained, consistent with the activity of telithromycin against the mutant. The shift of the C4 to A2058G C2 distance is expected given the presence of an amine group at the C2 position in G. In the methyl modifications, the C2 is not substituted and the shift to larger distances is indicative that the methylations weaken the interactions and that the conformation of 2611 relative to telithromycin is altered in these systems. The less pronounced broadening and shift of the distance distribution in DMAD is presumably a result of more restrained conformational sampling as suggested by the lower RMSF for both telithromycin and C2611 (**Figures S8, S9 in Text S1**). Overall, the results indicate that perturbation of the G2057 communication pathway impacts interactions with the methyl groups to the largest extent with the monomethylations.

**Figure 9:**
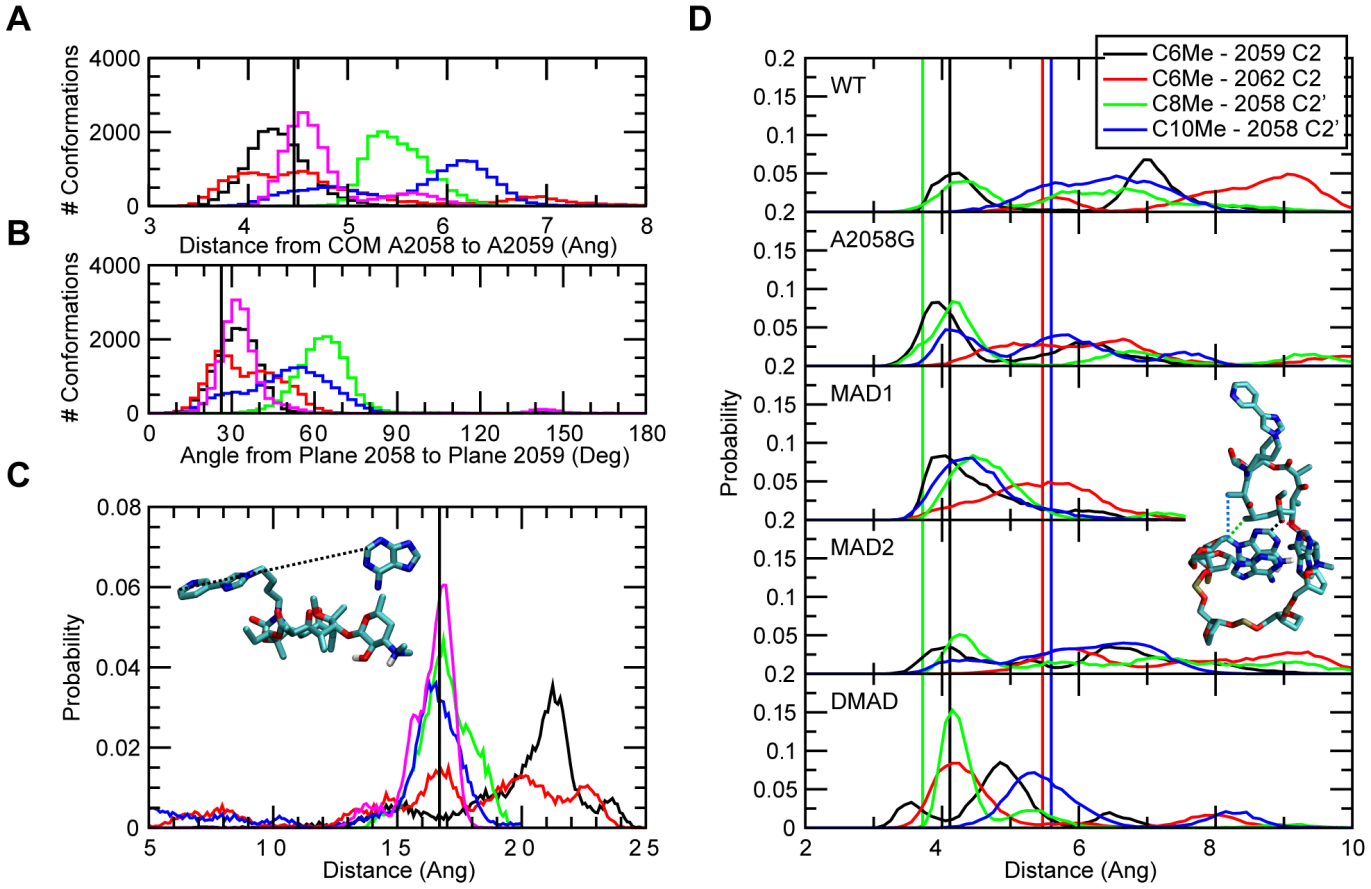
The number of conformations for WT, A2058G mutant and methyl modifications of (A) COM distances between base atoms of 2058 and 2059 and (B) angles between planes comprised by 2058 and 2059 base atoms. The probability distributions for all systems studied of the (C) telithromycin C26 to A2062 C2 distances and (D) telithromycin C6-methyl to 2059 C2 (black), C6-methyl to 2062 C2 (red), C8-methyl to 2058 C2′ (green), and C10-methyl to 2058 C2′ (blue). The crystal structure values are shown as vertical black lines in (A–C) and in (D) using the same color scheme, while distributions from the GCMC/MD simulations are shown as WT (black), A2058G (red), MAD1 (green), MAD2 (blue), and DMAD (magenta) in (A–C).

The third and final communication network considered was the A2059 pathway (**Figure 5**). This involves the covalent connectivity of A2058 to A2062, which is facilitated by stacking of A2058 and A2059 as well as a short interaction between A2059 C2 and A2062 N6 of 4 Å in the crystal structure. As performed above, stacking of 2058 to 2059 was analyzed based on the COM distance and angle distributions (**Figure 9a,b**). The stacking is well maintained in the WT and A2058G mutant, consistent with the activity of telithromycin in those species. The stacking is also well maintained in the double methylation while significantly perturbed in both the monomethylations. Concerning the A2059 C2 to A2062 N6 interaction, in the DMAD system this interaction is well maintained (**Figure S10 in Text S1**). Sampling of short distances also occurs in the other systems, but sampling of longer distances is present. Sampling of the longest distances occurs with WT and the A2058G mutant, which may contribute to the larger fluctuations of the ARM in those systems. This differential behavior could impact interaction of the nucleotides in the A2059 pathway with telithromycin. Nucleotides 2058, 2059, and 2062 come into VDW contact with the macrolactone of telithromycin. Shown in **Figure 9d** are probability distributions of selected distances between these bases and methyls extending from the macrocycle. All systems tend to sample short distances, with DMAD having a tendency to sample shorter distances to a larger extent than the other systems. Similar trends were observed for interactions between telithromycin's C9 and C16 keto groups with A2059 and A2062 as well as with a ribosomal Lys residue whose Nz atom is within 4 Å of one of the keto groups (**Figure S11 in Text S1**).

The final step in the A2059 communication pathway from 2062 to the A752-U2609 region involves indirect contacts. The region bordered by 2062, telithromycin, and 752/2609 is empty in the crystal structure and, thus, may be assumed to be filled with water, which likely contributes to the high flexibility of telithromycin's ARM. Such flexibility may allow for VDW contacts between 2062 and the ARM. Shown in **Figure 9c** are the distance distributions between C26 of telithromycin's pyridine and A2062 C2. The WT samples the longest distances indicating that the imidazole-pyridine moiety does not come close to A2062 and thus communication via this interaction does not occur. The A2058G mutant samples the widest range of distances, including some short distances (∼5 Å) that may perturb the heterocyclic ARM, potentially leading to the WC base pairing of A752-U2609 being altered. The methylated species mostly sample C26 and A2062 C2 distances similar to those observed in the crystal structure. This limited sampling is consistent with the RMS fluctuations of the ARM in these species. The exception is MAD2 that also samples shorter distances like the A2058G mutant and suggests that the MAD2 2058 modification may be propagated to A752-U2609 by a slightly different mechanism than the other methylated modifications. However, the lack of contact between A2062 and telithromycin's ARM heterocycle in the modifications indicates that the A2059 pathway is not as predominant as the G2057 pathway in the communication of 2058 mutation/modifications to A752-U2609.

### Conclusion

Altogether, our findings indicate that hydrogen bonding between 2′-OH and A2058 is important for telithromycin activity. Telithromycin maintains activity in the A2058G mutant via hydrogen bonds between the 2′OH and the WC hydrogen bonding groups of guanine, while decreased sampling of short 2′-OH to A2058 distances in the monomethylated species contributes to their lowered activity. In addition, the mutation/methylations are predicted to alter base stacking interactions with 2057 and to a lesser extent with 2059. These perturbed stacking interactions are communicated to more remote regions of the ribosome that comprise the telithromycin binding pocket thereby contributing to changes in telithromycin's activity in the methylated species. These changes occur through three potential pathways identified in this study: the telithromycin pathway, the G2057 pathway and the A2059 pathway. Analysis indicates the G2057 pathway leads to the largest conformational changes, including alterations of interactions of nucleotides 2058, 2610 and 2611 with methyl groups on telithromycin and, importantly, perturbation of A752-U2609 base pairing and interactions of those bases with the ARM of telithromycin.

In the context of telithromycin activity in wild type and A2058-methylated ribosomes, studies suggest that high levels of A2058-dimethylated ribosomes are required to confer telithromycin resistance [25]. Minimum inhibitory concentration (MIC) values for telithromycin are only marginally increased (∼4-fold) in monomethylated ribosomes as opposed to 256-fold for erythromycin [23]. The ability of telithromycin to maintain activity against monomethylated ribosomes has been proposed to result from imidazole-pyridine to A752-U2609 stacking interactions that mitigate the effects of disrupted 2′-OH – A2058 hydrogen bonds [15], [26]–[28] and our findings coincide with this assessment. While hydrogen bonding does occur between telithromycin's imidazole and 2609 in MAD1, the ARM and 752–2609 maintain distances and plane angles that are indicative of stacking in a large number of conformations and 752–2609 WC distances are sampled with a higher probability than non base-paired or non-stacked distances.

Recently, Melman and Mankin [58] suggested that disruption of the 2′-OH – A2058 N1 hydrogen bond was not the major reason that telithromycin activity is reduced in A2058-dimethylated ribosomes. They found that removal of the 2′-OH from telithromycin did not increase MIC values to the extent that Erm(A) expression did. In other words, disruption of the hydrogen bond was not the predominant explanation for loss of telithromycin activity. They propose that the major explanation for reduced telithromycin activity is more likely that the overall structure within the binding site is perturbed upon the introduction of methyl groups onto A2058 as a result of nonbonded interactions between the methyl groups and nearby crystallographic waters. The exocyclic A2058 N6 is within 4 Å of water molecules that are coordinated to Mg^+2^ that chelates the phosphate groups on G2056 and G2057. Accordingly, the authors suggest that interactions between the N6-methyl groups and water molecules lead to structural changes in the dimethylated ribosomes that reduce telithromycin activity. The results presented here further suggest that the conformation of RNA in the macrolide binding pocket is perturbed in both the 2058 mutant and modified ribosomes. However, the present results indicate that this is due to altered base stacking interactions with 2057 and 2059 that are propagated to other bases in the G2057 and A2059 pathways (**Figure 5**), respectively. To test the hypothesis of Melman and Mankin [58], the distance between the exocyclic N6 of A2058 and the Mg^+2^ ion were compared in the WT and mutant/modified systems (O6 in A2058G mutant). In all the systems except the mutant, the interaction is shifted by >1 Å indicating that the crystal structure distance is slightly too short (**Figure S12 in Text S1**). The shift to shorter distances for the A2058G mutant is expected given that the exocyclic N6 amine of A is replaced by a keto group in G. The distribution of distances show a high degree of overlap between WT and DMAD indicating that the relative position of the magnesium ion does not change upon introduction of two methyl groups onto A2058. Moreover, the distribution for DMAD is sharper suggesting that dimethylation restricts the conformational sampling of the 2058 N6 to Mg^2+^ distance.

A particularly interesting result is the flexibility of the ARM in WT and the A2058G mutant versus that occurring in the methylated species. The fluctuations of the ARM are significantly higher than the rest of telithromycin in the WT crystal structure, corresponding to an average RMS fluctuation of 1.55 Å at 100 K over the non-hydrogen atoms in the heterocycles in the ARM. Thus, while stacking of the ARM with A752-U2609 is occurring to some extent, this is clearly a dynamic region of the system. The flexibility of the ARM is consistent with kinetic studies of telithromycin binding to the *E. coli* ribosome [59], which show that telithromycin's shift from a low to a high-affinity state results from reorganization of the ARM, and may also explain the various conformations of the alkyl-aryl ARM seen in different crystal structures [15], [26], [38]. Given that this flexibility is large in both the WT and A2058G mutant and significantly lower in the methylated species, it suggests a scenario where entropic contributions associated with the flexibility of the ARM, and possibly the surrounding environment, makes a favorable contribution to binding. Upon methylation the mobility of the ARM is decreased, thereby contributing to a decrease in binding affinity. However, this is due to increased hydrogen bonding between the ARM and the A752 and U2609 nucleotides, interactions that could be exploited to improve the binding affinity. Essentially, a favorable entropic contribution to binding is being switched to a potentially favorable enthalphic contribution. Indeed, this appears to be the case with the recently published analog, solithromycin, in which the ARM imidazole-pyridine moiety was replaced with a triazolyl-aminophenyl group allowing for additional hydrogen bonding with 752 as well as nearby 748 [60]. Namely, the hydrogen bonds lead to a favorable enthalpic contribution while decreasing the favorable entropic contribution which is consistent with the experimentally observed decrease in the RMS fluctuations from 1.5 to 1.0 Å for the ARM non-hydrogen atoms upon going from telithromycin to solithromycin. While solithromycin shows improved activity compared to telithromycin against Erm-based modifications, which may be attributed to this hydrogen bonding, the presence of a C2-fluoro group not present on telithromycin may also contribute to improved binding. The C2-fluoro group appears to form a favorable (2.7 Å), hydrophobic interaction with C2611 thereby complicating interpretation of the contribution of ARM hydrogen bonding to affinity.

The results presented herein may be used to suggest modifications to telithromycin, or future ketolides, that could improve its binding to Erm-methylated ribosomes. These include modifying telithromycin's ARM to 1) engage in hydrogen bonding interactions with 752, 2609 and adjacent nucleotides, leading to an enthalpic contribution to binding or, on the other hand, 2) decrease the potential for such hydrogen bonding and/or increase the conformational flexibility in the ARM, leading to an entropy gain. Another region for potential improvement of binding against the methylated species involve modifying the macrolactone of telithromycin. The present calculations indicate the telithromycin methyl-base interactions to be longer in the modified ribosome. Accordingly, modifications to telithromycin that add steric bulk to the methyl groups nearby 2058, 2057, and 2611 may enhance VDW interactions with the ribosome that may also gain from an increased hydrophobic contribution to binding. Such modifications will be guided by previous work showing that ketolides bearing C2 groups larger than F are devoid of activity [61] as well as the results with solithromycin [60] showing that C2-fluorination may increase ketolide activity against Erm-based modifications.

## Methods

Calculations were performed with the program CHARMM, version C36a6 [62] and the CHARMM additive force field including the protein with the CMAP correction [63]–[65], nucleic acid [66]–[69], carbohydrate [70]–[75], and CGenFF [76] parameters and the TIP3P water model [77]. Coordinates were obtained from the protein database (PDB ID 3OAT [38]), with hydrogens added using the HBUILd facility in CHARMM. Since only the region around the telithromycin binding site was of interest, residues without one or more atoms within 40 Å of the center of system, as defined by the center of mass of telithromycin, were deleted. The system was then overlaid with a 28 Å water sphere and any waters bearing an oxygen within 2.8 Å of a solute non-hydrogen atom were deleted. Mutants were generated using this truncated system, with patches applied to A2058 in order to generate the A2058G, N6-monomethyl, and N6,N′6-dimethyl A2058 variants. Initial guess parameters for the base methylations were obtained from ParamChem [78], [79]. Bond lengths, angles, and dihedrals specific to the mutations were optimized via comparison to quantum mechanical (QM) geometries, water interactions, molecular vibrations and dihedral potential energy scans. Details of the parametrization are included in the Supporting Information (Text S1).

MD simulations were performed using a stochastic boundary-based approach [80]–[84] in which three regions within the sphere were defined. Nucleotides and residues containing one or more atoms within 28 Å comprise the dynamic region, those not in the dynamic region containing one or more atoms within 34 Å comprise the buffer region, and the remaining atoms based on the 40 Å cutoff represent the constrained outer reservoir region. Nucleotides 732, 696, 2458 and 2459 are found along the border of the buffer and reservoir regions, and were manually assigned to the reservoir region; nucleotides 766 and 1324 border the dynamic and buffer regions and were assigned to the buffer region. All crystallographic Mg^+2^ ions within the 40 Å radius sphere were included in the simulation system, yielding a total of 82 ions. Atoms within the reservoir region were fixed for all calculations, while varying harmonic restraints were used on atoms within the buffer and dynamic regions as described below. Water was maintained within the sphere using a spherical, quartic restraining potential as implemented in the MMFP [85] module of CHARMM using a 1 kcal/mol/Å force constant and offset parameter (P1) of 2.5 that was applied to the water oxygen atoms.

The entire system was first subjected to 250 steps of steepest descent (SD) [86] minimization with a harmonic restraint of 5 kcal/mol/Å on non-hydrogen atoms within the dynamic region and a mass-weighted harmonic restraint of 10 kcal/mol/Å on non-hydrogen atoms within the buffer region, followed by 250 steps of Adopted-Basis Newton Rhapson (ABNR) [86] using the same restraints. Equilibration consisted of two phases of Grand Canonical Monte Carlo/Molecular Dynamics (GCMC/MD), which is implemented within the MC module in CHARMM [87]. The MC module has been described in detail elsewhere [52], [87]–[89], thus the details of the general methodology will only briefly be addressed as they pertain to the simulations presented here.

A total of 8997 water molecules were used for the bath of water molecules accessible to the GCMC move set. This number was determined by calculating the number of water molecules that would result in a spherical volume of 35 Å radius with density 0.0334 molecules per Å^3^, then multiplied by 1.5 to guarantee that an excess of water molecules were available. The move set was comprised of rigid body translations, rigid body rotations, as well as insertion/deletions that were performed using an excess chemical potential of −5.8 kcal/mol. All moves were equally weighted. The GCMC water pool includes the waters overlaid on the system as described above and the additional water molecules that were placed in a single coordinate set slightly offset from the heterocyclic ARM of telithromycin. All crystallographic waters were set as active throughout the entire simulation. The GCMC waters were initially set as inactive, thereby removing them from all calculations. Each GCMC/MD cycle is defined as 10,000 MC steps and 10,000 MD steps. Initiation of each MD cycle required assignement of the velocities as those from the previous MD cycle are invalidated by the insertions and deletions during each GCMC cycle. Reassignment of the velocities was done by employing the Langevin integrator for the MD simulations (see below). During the GCMC steps, waters can undergo moves as defined in the move set, in which particle insertions were set to be blocked by all active atoms including hydrogens. The initial hydration phase consisted of 20 GCMC/MD cycles using 5 kcal/mol/Å harmonic restraints on non-hydrogen atoms within the dynamic region and a 10 kcal/mol/Å mass-weighted harmonic restraint on non-hydrogen atoms within the buffer region. For equilibration, the list of active GCMC waters was reset and the harmonic restraints were removed for atoms within the dynamic region and reduced to 2 kcal/mol/Å on non-hydrogen atoms within the buffer region. Iterative GCMC/MD cycles were performed until the number of waters reached adequate convergence (approximately 5 ns cumulative dynamics simulation time). Once the number of waters reached convergence, production consisted of GCMC/MD for a total dynamics simulation time of 25 ns using the same restraints/constraints. Only coordinates from production GCMC/MD were used for analysis. Additional GCMC/MD simulations were performed in which longer MD sampling was performed per cycle (ie. 100, 250, 500 and 1000 ps) to test convergence of the method with respect to water insertions (see below and SI). As described in the SI, these different simulations of 25 ns sampling each were ultimately combined for the final analysis yielding a total of 150 ns of cumulative MD sampling for each of the studied systems.

MD simulations were performed using Langevin dynamics [90], [91] at 298 K with a friction coefficient of 5/ps. We note that the use of Langevin dynamics (LD) is not limited to cases without explicit solvent and in fact is often used in explicitly solvated systems as a temperature control, particularly with the program NAMD [92], as is the case here. While the explicit presence of water molecules accounts for the frictional effects on the system, the use of LD is justified because the friction coefficient of 5/ps used is much less than that appropriate to mimic the viscosity of water (ie. ∼60/ps). SHAKE [93] was applied to covalent bonds involving hydrogens, and a 2 fs integration timestep was used with the “leapfrog” Verlet integrator [94]. Nonbonded lists were updated heuristically during dynamics with a cutoff of 16 Å, the forces truncated at 12 Å, and a switching function applied to the forces from 10 to 12 Å for both electrostatic and van der Waals energy terms [95]. Coordinates were saved every 10 ps for analysis. Hydrogen bonds were considered present if the hydrogen donor and acceptor atoms came within 2.4 Å [96] and have an occupancy greater than 10%.

## Supporting Information

Text S1 **Parametrization, validation of methodology, and supporting material.** Parametrization details, validation of methodology, Figures S1, S2, S3, S4, S5, S6, S7, S8, S9, S10, S11, S12, Tables 1–7, and additional references.
(PDF)
