# Supplementary material for: Impact of Ribosomal Modification on the Binding of the Antibiotic Telithromycin Using a Combined Grand Canonical Monte Carlo/Molecular Dynamics Simulation Approach
Source: PLoS Comput Biol. 2013 Jun 13;9(6):e1003113. doi: 10.1371/journal.pcbi.1003113 (PMC3681621; doi:10.1371/journal.pcbi.1003113)
Supplement: Text S1 — Parametrization, validation of methodology, and supporting material. Parametrization details, validation of methodology, Figures S1, S2, S3, S4, S5, S6, S7, S8, S9, S10, S11, S12, Tables 1–7, and additional references. (PDF) [file pcbi.1003113.s001.pdf]

Supporting Information for Impact of Ribosomal Modification on the Binding of the Antibiotic Telithromycin using a Combined Grand Canonical Monte Carlo/Molecular Dynamics Simulation Approach

*Meagan C. Small†, Pedro Lopes†, Rodrigo B. Andrade‡, Alexander D. MacKerell, Jr.†**

† Department of Pharmaceutical Sciences, University of Maryland, Baltimore, MD 21201

‡ Department of Chemistry, Temple University, Philadelphia, PA 19122

<u>Parametrization details for methylated A2058</u>

Parametrization of the N6-mono- and N6,N6'-dimethyl adenine moieties followed the CGenFF optimization protocol.[1] For efficiency, the ribose and phosphate groups were removed and N9 was capped with a hydrogen atom. Initial parameters were obtained from the CGenFF program, version 0.9.1, via the ParamChem interface (available online).[2,3] Mol2 files were generated using MOE (Molecular Operating Environment[4]). Charges obtained from ParamChem were replaced by standard charges from the CHARMM36 nucleic acid force field[5-8] for all atoms with the exception of the exocyclic N6 and N9, the latter of which whose

ParamChem "guess" charge was maintained because of the capping hydrogen atom. Structures were initially generated in CHARMM[9] using initial guess parameters, then the geometry optimized at the quantum mechanical (QM) MP2 level of theory with the 6-31G* basis set and tight optimization with the program Gaussian03.[10] Charges were first optimized by comparison of QM and (CHAR)MM interaction energies with water. QM interaction energies were calculated using Gaussian03, with the Hartree-Fock level of theory and 6-31G* basis set as previously described.[1,8] Because methylation occurs at the exocyclic N6 and presumably would have little effect on the charges of other atoms, the charges for all other adenine atoms were maintained and only the methyl carbon and N6 charges were optimized. Hydrogens of the exocyclic methyl group were maintained at 0.09. **Table S2** and **S3** show the interaction energies and distances for N6-mono and N6,N6'-dimethyl adenine and water in various geometries, respectively. Only the most favorable geometries were selected as target QM data.

Bond lengths and angle parameters for the exocyclic methyl groups were optimized using the QM target geometry. The N6-monomethyl adenine molecule was optimized first and the parameters transferred to the dimethyl analog. In general, the CGenFF parametrization protocol permits deviations of 3° for the angles and 0.03 Å for the bonds. However, the difference in the angle N1-C6-N6 was found to be 3.7°. The associated parameter belongs to the existing parameters within the nucleic acid force field and not subject to optimization, therefore the slight increase in deviation was deemed acceptable. For N6,N6'-dimethyl adenine, parameters for the bond and angle lengths were initially directly transferred for the monomethyl analog. However, because the agreement with QM data for the bonds and angle was poor, the second methyl group was defined as a unique atom type from the first methyl carbon, allowing the dimethyl analog to be specifically parametrized separately. Again, deviations between the

QM and MM bond and angle lengths for the atoms of interest were found to be less than 3° for the angles and 0.03 Å for the bonds, with the exception of N1-C6-N6 which was found to be 4.7°, see **Figure S1** for atom numbering.

Bond and angle force constants were optimized by comparison of vibrational spectra for the QM and MM geometries, in which QM frequency values were scaled by 0.943.[11] Vibrational spectra were calculated via the MOLVIB module in CHARMM. Frequencies and contributions of the internal degrees of freedom were compared for both the QM and MM geometries and bond/angle force constants were manually optimized until the vibrational spectra were in good agreement, **Table S4** and **S5** for the mono- and dimethyl adenine molecules.

The force constants for the dihedral angles C5-C6-N6-C7A and C5-C6-N6-C7B, where C7A/B represent the methyl carbons, were optimized using potential energy surfaces (PES). QM scans were performed with Gaussian03[10] from 0° to 180° in 12 steps of 15° for each targeted dihedral. CHARMM was utilized to generate the MM PES. The relative energies at each scan point were compared between QM and MM and the force constants and phases were manually changed until good agreement was achieved (**Figure S2**). Phases were constrained to 0° or 180° as per the CGenFF convention. During the optimization emphasis was placed on the low energy regions of the PES as these are sampled during the MD simulations.

Because optimization of the dihedral yielded high potential energy barriers for this dihedral, it was necessary to consider two separate conformations for the N6-monomethyl mutant, one in which the methyl group was oriented cis to the N1 atom and the other in which the methyl is trans. QM and MM energies were calculated for both orientations, in which the methyl group oriented cis to N1 was designated MAD1 and trans to N1 as MAD2. **Table S6** shows the QM and MM energies for both orientations. QM energies were obtained using

Gaussian03[10] using the MP2 level of theory and 6-31G* basis set and tight optimization. MM energies were obtained in CHARMM using the optimized parameters aforementioned and performing an energy minimization in vacuo with conjugate gradient for 200 steps and and Newton-Raphson minimization for 50 steps with a tolerance of 0.00001 using infinite non-bond cutoffs.

System Set-up and Equilibration

Simulations of the entire ribosomal complex are computationally expensive- e.g. solvating the complex within a box of water would result in a system of over 1 million atoms.[12,13] Since only atoms pertaining to the telithromycin binding site are of interest for the present study, a stochastic boundary approach[14-18] is utilized whereby atoms of interest encompassing the telithromycin binding site "see" an external field that is representative of atoms outside of the region of interest. Furthermore, because of the buried nature of the binding site and the fact that it is inaccessible to the bulk region, a Grand Canonical Monte Carlo-Molecular Dynamics (GCMC/MD) approach is employed.[19,20] GCMC/MD allows for the number of waters to fluctuate during the course of the simulation resembling an open system at a constant chemical potential. Hence it is utilized here as a means to robustly determine the degree of hydration within the telithromycin binding site during the simulation. GCMC/MD is divided into two stages. In the first (GCMC) waters are inserted, deleted, rotated, or translated according to a Metropolis acceptance criterion[21] that is dependent on the excess chemical potential of water and the change in energy between the putative position and existing position. The second stage (MD) allows for subsequent relaxation of the system's dynamic regions, including the newly placed waters. These stages are repeated iteratively until the number of waters within the

system reaches convergence, with this stage of the simulation considered equilibration. All the systems (i.e. WT, mutant, and modifications) were treated in this manner and found to reach convergence similarly. Shown in **Figure S3** are the number of waters as a function of simulation time for all the systems studied. The number of active GCMC waters approaches convergence by 5 ns (a total of 250 GCMC/MD cycles) and this portion of the trajectory is considered equilibration. At this point, the running average of GCMC waters within the 35 Å sphere was determined to be 2115 with a standard deviation of 2 water molecules for WT. Equilibration of the system was confirmed by root mean square deviation analysis (**Figure S4**), which shows that the ribosome (RNA and protein) is stable and has relaxed around the newly placed waters by 5 ns. Though the dynamics is not continuous as in standard molecular dynamic simulations, because the positions of the system (namely, RNA, protein, and ligand) are unchanged during the GCMC portion, it is treated as continuous for the purposes of analysis. From the RMSD, the deviation is less than 2.5 Å in all cases and was assumed to be adequately converged by 5 ns such that the remaining 25 ns was considered production and was used for analysis. As a check, the potential energy as a function of cycle was calculated and was also found to stabilize by 250 cycles (or 5 ns dynamics time, **Figure S5**). Following equilibration, production GCMC/MD is performed which allows the systems to sample a range of conformations and degrees of hydration.

<u>Molecular dynamics using longer MD simulations during each GCMC/MD iteration.</u>

The convergence of the GCMC/MD simulations was confirmed by running multiple simulations using longer MD during the GCMC/MD cycles. The number of MC steps remained unchanged at 10,000. Starting from the coordinates obtained after the initial hydration phase

(which involves 20 cycles of GCMC/MD with restraints/constraints as defined in the methods), these additional simulations consisted of an initial 100 cycles of GCMC/MD using 10,000 steps-20 ps MD per cycle for a total of 2 ns. At this point the majority of GCMC waters were inserted. The coordinates were then used to start 4 separate simulations in which the number of MD steps was increased to 25,000 steps, 50,000 steps, 250,000 steps, or 500,000 steps for total dynamics simulation times of 50 ps, 100 ps, 500 ps, and 1 ns per cycle, respectively. A fifth GCMC/MD simulation was also performed using the same number of MD steps per cycle (10,000 steps/20 ps) so as to test the original simulation protocol. GCMC/MD simulations were continued for 28 ns bringing the total dynamics time to 30 ns with the last 25 ns used for analysis. This procedure was applied to all the WT and A2058 mutant/modified systems.

Analysis showed the number of waters inserted during GCMC to be correlated to the length of the MD, **Figure S6**. Thus, the longer MD simulations appear to allow for additional relaxation of the structure allowing for a higher number of waters to be inserted overall. To determine whether this effect would alter the dynamics within the macrolide binding pocket, the number of waters within 10 Å of telithromycin was counted over the last 25 ns and averaged for each simulation (**Table S7**). Waters were counted if the oxygen atom resided within 10 Å of the center and the final snapshot of each trajectory was used to determine the water coordinates. The number of waters was found to be similar irrespective of the length of MD and therefore not likely to affect the dynamics within the pocket. RMSD analysis was also performed for the varying MD simulations and found to be within 2.5 Å (not shown).

The impact of the length of the MD simulations, as well as convergence of the sampling, was also assessed by analyzing probability distributions of the telithromycin 2'-OH to N1/N6 A2058/A2059 distances for the wild-type and mutant simulations (**Figure S7**). Overall, good

agreement was observed between the simulations using different duration MD simulations indicating that the ensemble of conformations sampled during the different simulations were sampling similar, though not identical regions of conformational space. For this reason, the conformations from all of the simulations were combined and used for analysis, which yielded a total of 150 ns cumulative sampling time for each of the studied systems.

Root mean square fluctuations to determine conformational flexibility

Root mean square fluctuations (RMSF) were calculated for RNA and protein within 10 Å of telithromycin as well as telithromycin to determine whether A2058 modification impacts the conformational flexibility of the macrolide binding pocket or of the macrolide. RMSF values were calculated for non-hydrogen atoms of the nucleotide (including base, sugar, and phosphate group). B-factors are also shown for completeness and are calculated by averaging over the atomic B-factors for each nucleotide.

Figure S8 shows that RNA and protein are in general more conformationally flexible in the WT ribosome. Moreover, **Figure S9** indicates that the arm of telithromycin is more flexible in the WT and A2058G mutant and that this increased flexibility coincides with the B-factors from the crystal structure as discussed in the text.

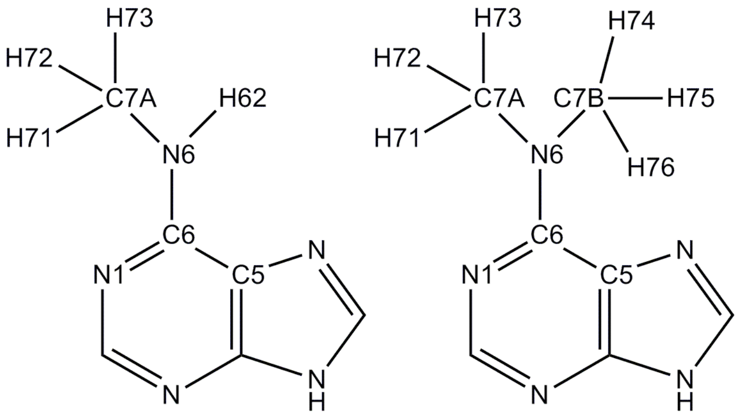

Figure S1. 2D representation of N6-monomethyl adenine (left) and N6,N6'-dimethyladenine (right) showing the numbering scheme pertaining to the parametrization.

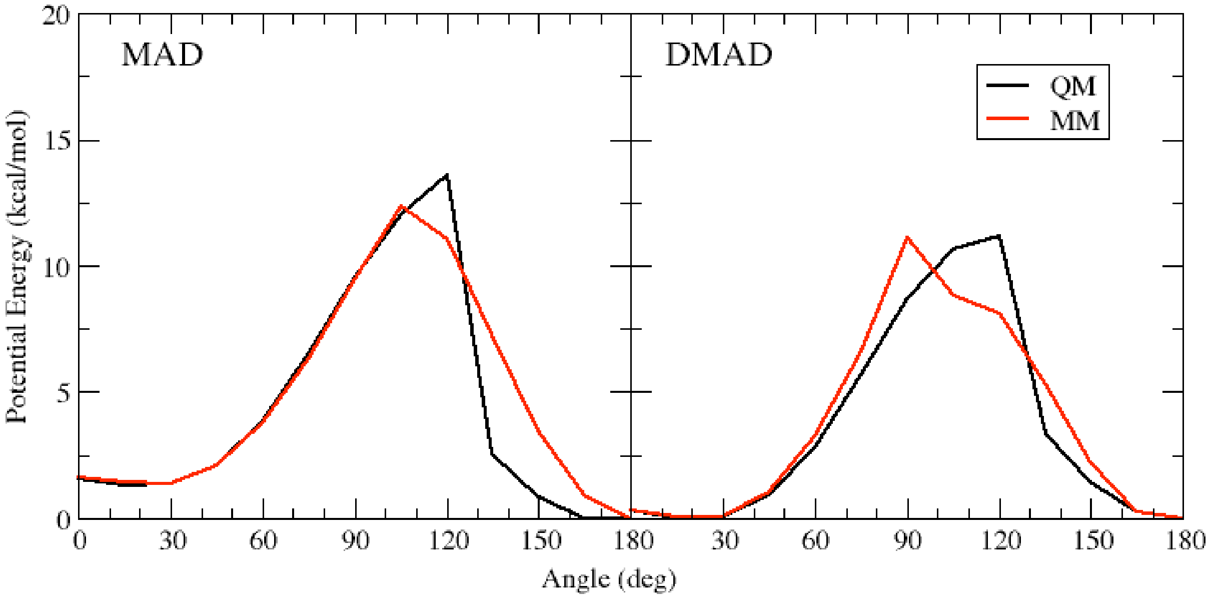

Figure S2. Potential energy scan for (left) N6-monomethyl adenine for the dihedral C5-C6-N6-C7A and (right) N6,N6'-dimethyl adenine dihedral C5-C6-N6-C7B, where C7A/B represent the exocyclic methyl carbons as shown in Figure S1.

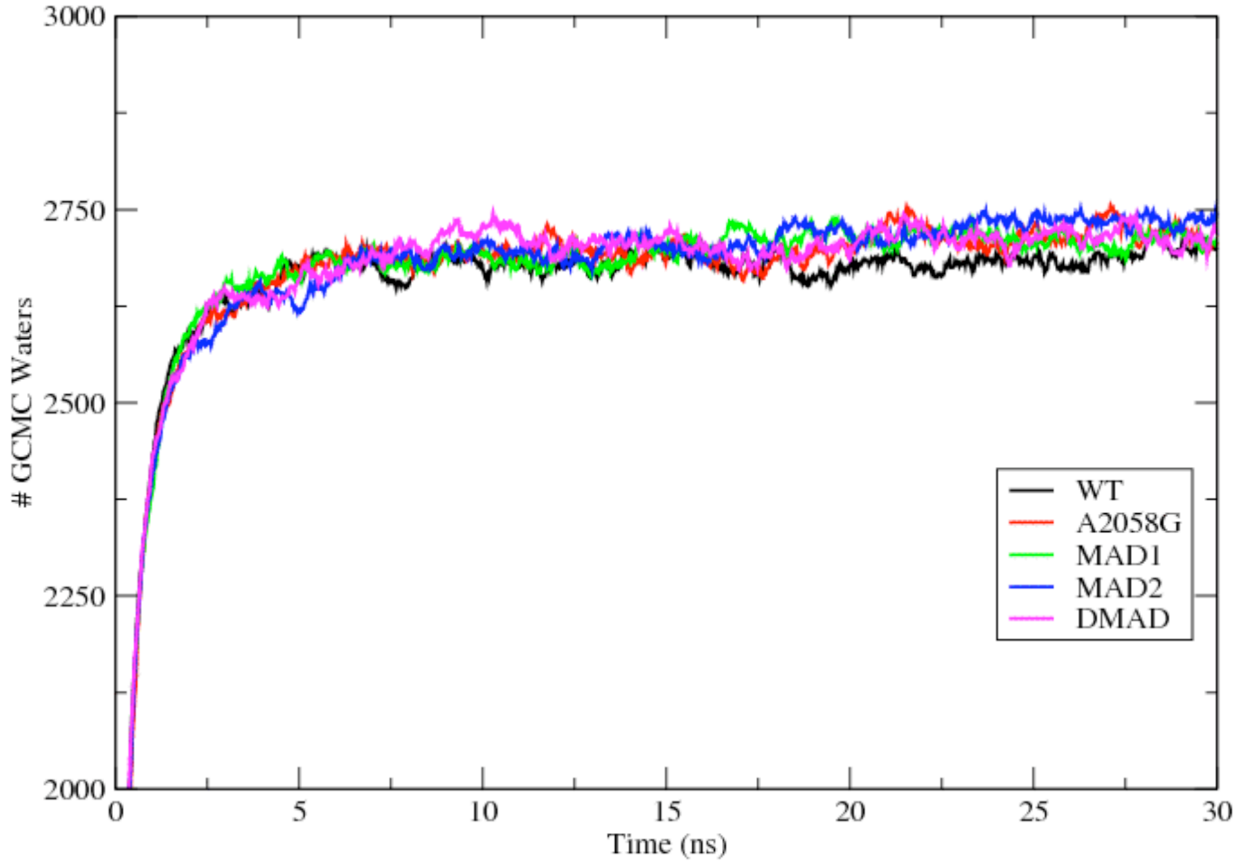

Figure S3. The number of GCMC waters as a function of dynamics simulation time for each system studied. Number of GCMC waters were those present at the start of MD during each cycle. WT=wild-type (black), A2058G=A2058 mutated to G (red), MAD1/MAD2= the N6-monomethyl adenine with the methyl cis (green) and trans (blue) to the N1 atom, DMAD= N6,N6'-dimethyl adenine (magenta).

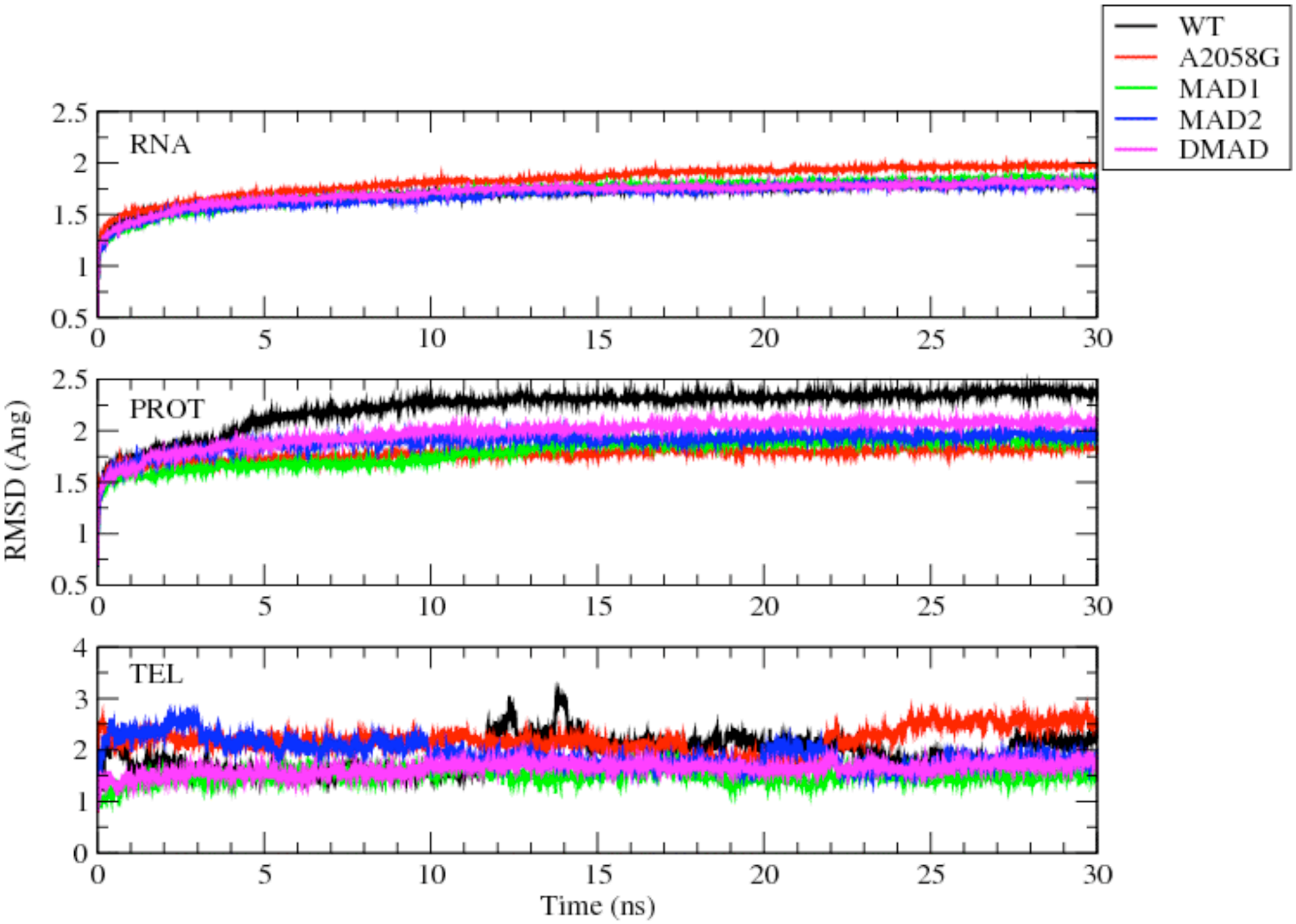

Figure S4. The RMSD (in Ang) for RNA (top panel), protein (middle), and telithromycin (bottom) for the WT and mutant/modifications over the 30 ns GCMC/MD simulation. WT is shown in black, A2058G in red, MAD1 in green, MAD2 in blue, and DMAD in magenta.

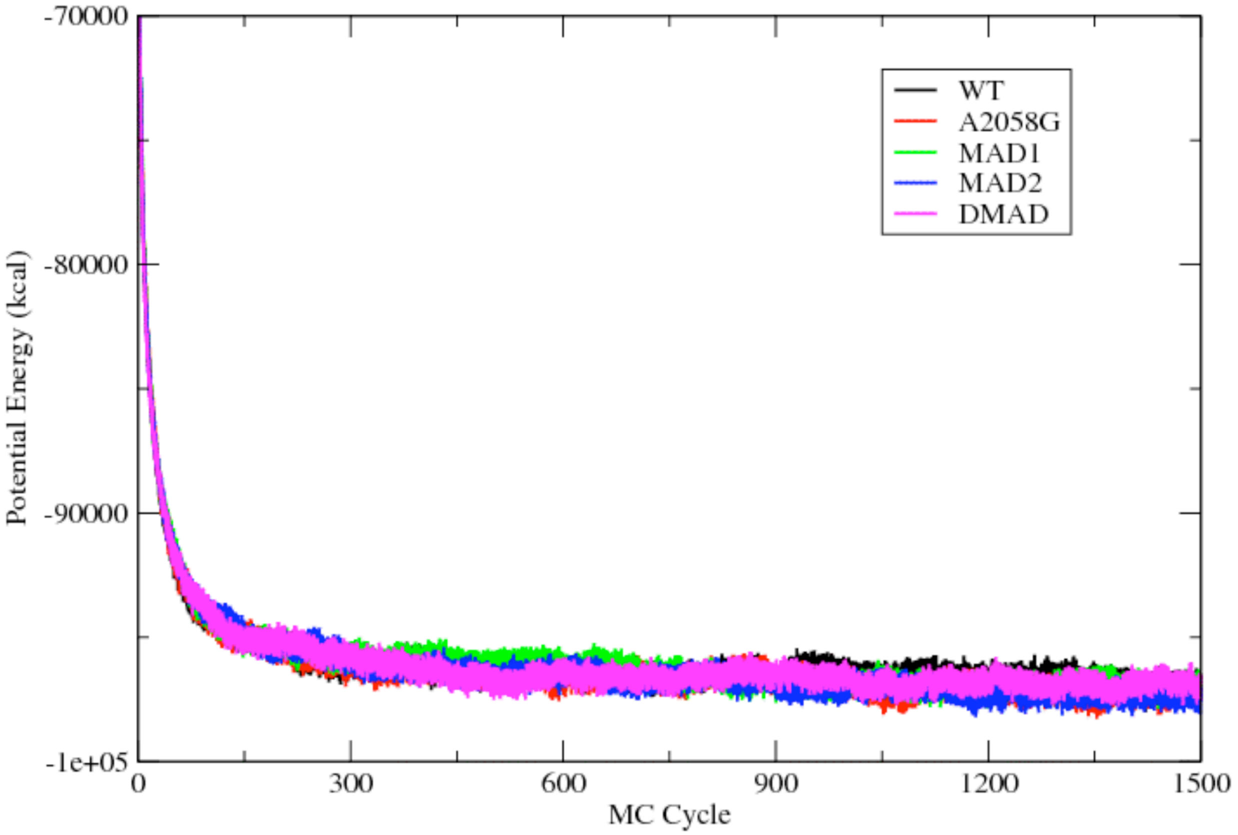

Figure S5. The potential energy as a function of cycle for the WT and mutant/modifications over the 30 ns GCMC/MD simulation. 1500 cycles is equivalent to 30 ns dynamics simulation time. WT is shown in black, A2058G in red, MAD1 in green, MAD2 in blue, and DMAD in magenta.

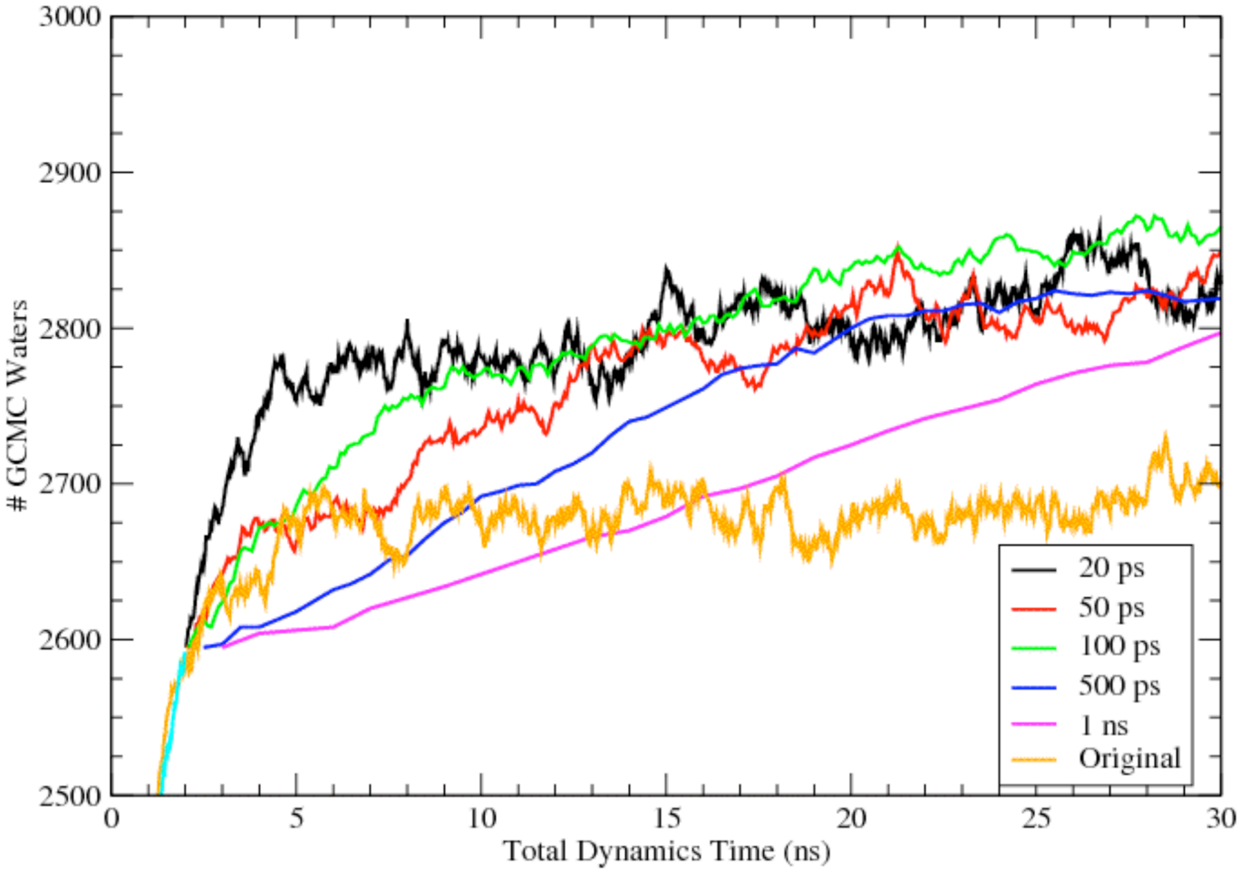

Figure S6. The number of GCMC waters as a function of time for varying lengths of MD performed to check for convergence. Original refers to the GCMC/MD simulation in which all 1500 cycles were run continuously using 10,000 MC steps and 20 ps MD. The 20, 50, 100, 500 ps, and 1 ns lines refer to the GCMC/MD simulations used to test for convergence and were performed by first running 100 cycles of GCMC/MD (10,000 MC steps/20 ps MD), then using the final snapshot to start 5 separate GCMC/MD simulations with varying MD steps as described in the text. For this reason, the number of GCMC waters in the latter GCMC/MD simulations are shown starting at 2 ns with the number of GCMC waters during the first 2 ns shown in cyan. The Original starts at the origin.

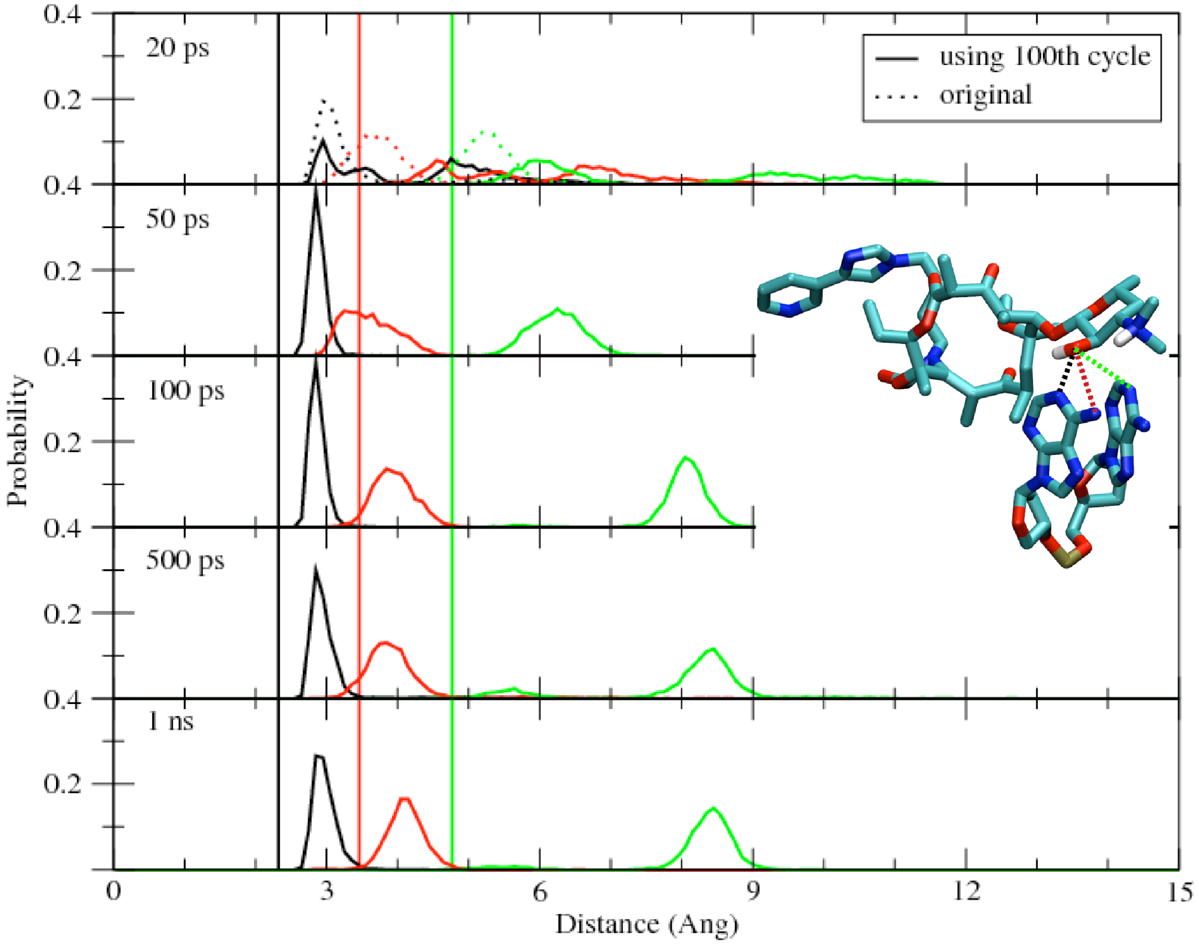

Figure S7. The probability distributions of the telithromycin 2’OH to N1, N6 (A2058) and N6 (A2059) for the various lengths of molecular dynamics simulations performed to determine convergence. The 2'-OH – N1 (A2058) is shown in black, 2'-OH to N/O6 (A/G2058) in red, and 2'-OH to N6 (A2059) in green. Corresponding crystal structure distances are illustrated as vertical lines using the same color scheme. The two GCMC/MD simulations obtained with 20 ps MD are shown. Original refers to the GCMC/MD simulation in which all 1500 cycles were run continuously using 10,000 MC steps and 20 ps MD. The second simulation in which the coordinates were obtained from an initial 100 cycle GCMC/MD simulation is designated as such and is described in the text.

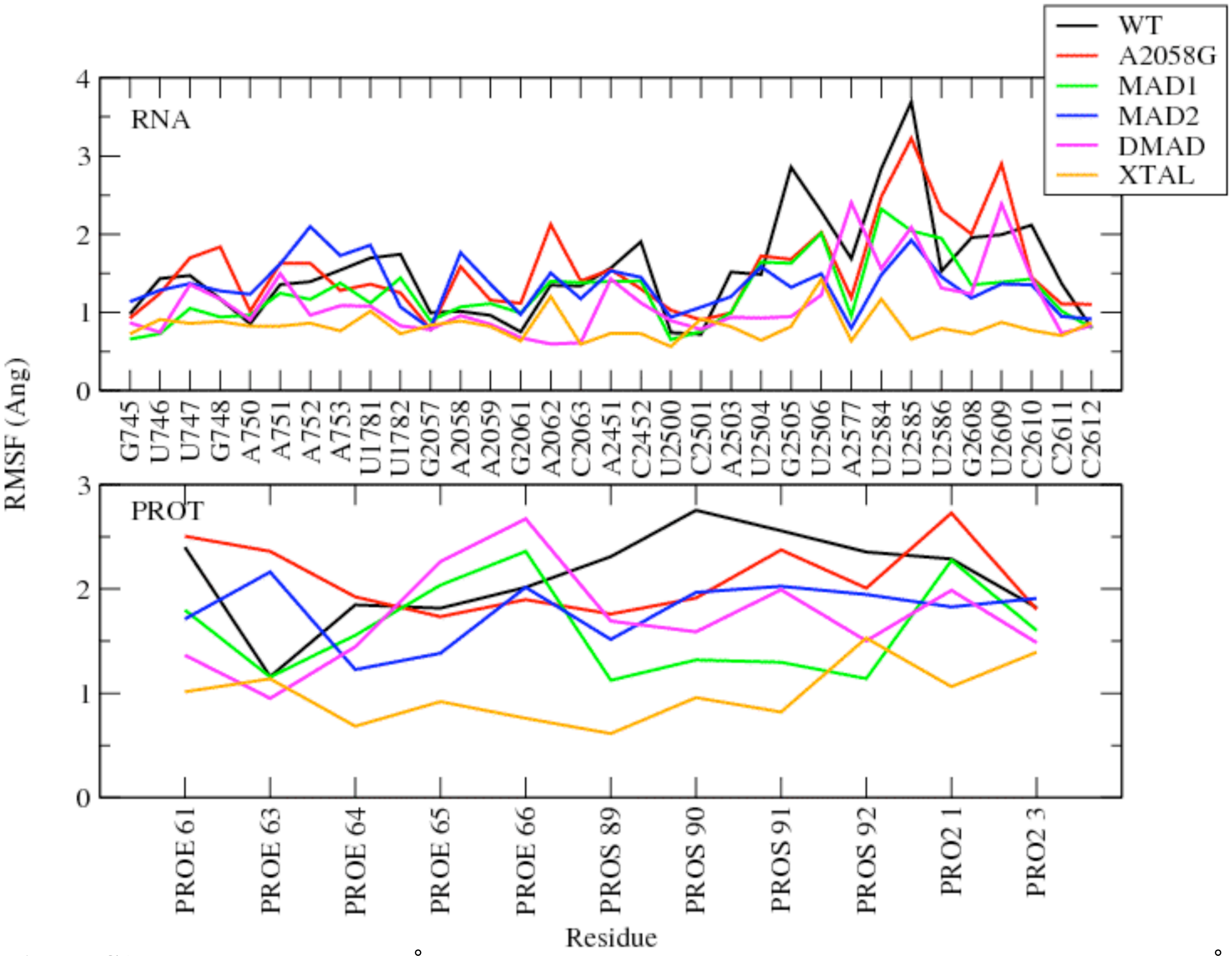

Figure S8. RMS Fluctuations (Å) for RNA (top panel) and protein (bottom panel) within 10 Å of telithromycin in the WT and mutant/modifications. WT is shown in black, A2058G in red, MAD1 in green, MAD2 in blue, DMAD in magenta, and crystal structure values in orange. RMSF values reported are for the entire nucleotide or residue (including backbone).

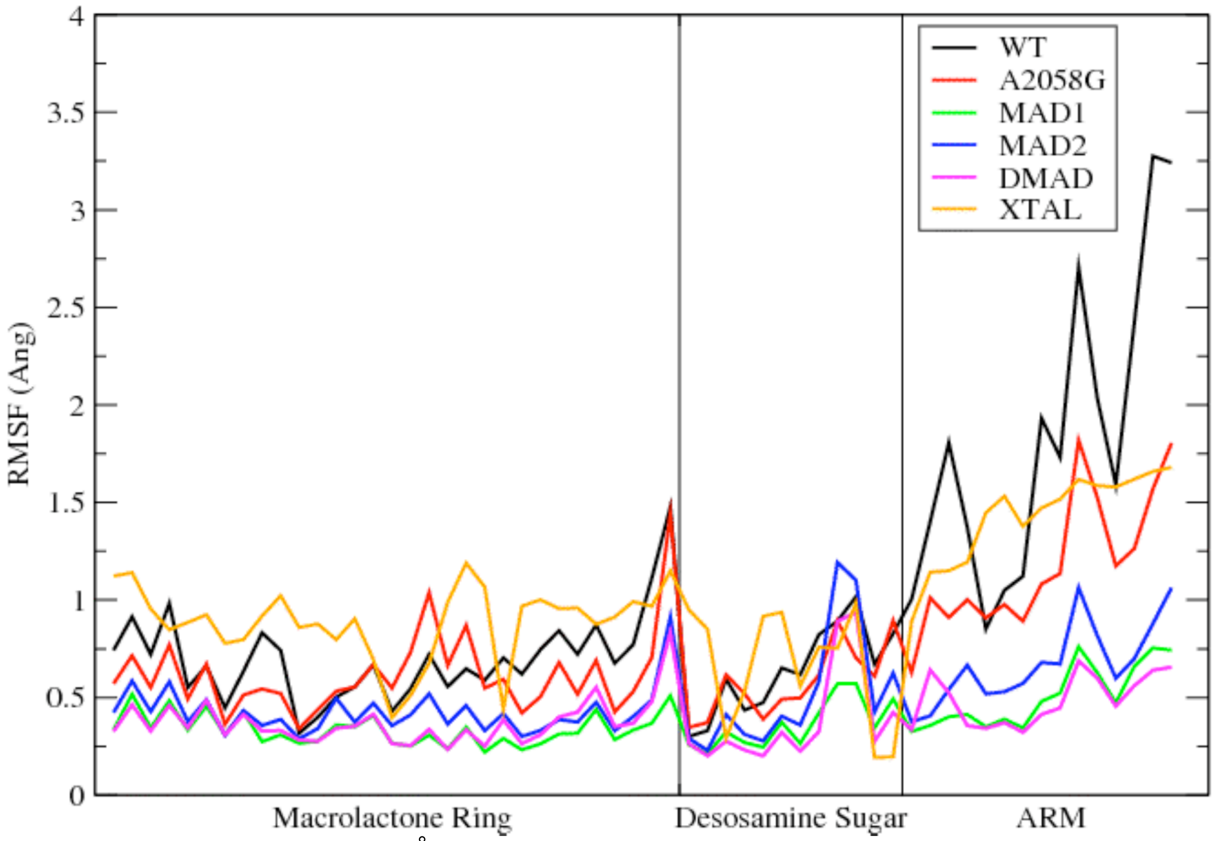

Figure S9. RMS Fluctuations (Å) for telithromycin in the WT and mutant/modifications. WT is shown in black, A2058G in red, MAD1 in green, MAD2 in blue, DMAD in magenta, and crystal structure values in orange. RMSF values reported are for non-hydrogen atoms only.

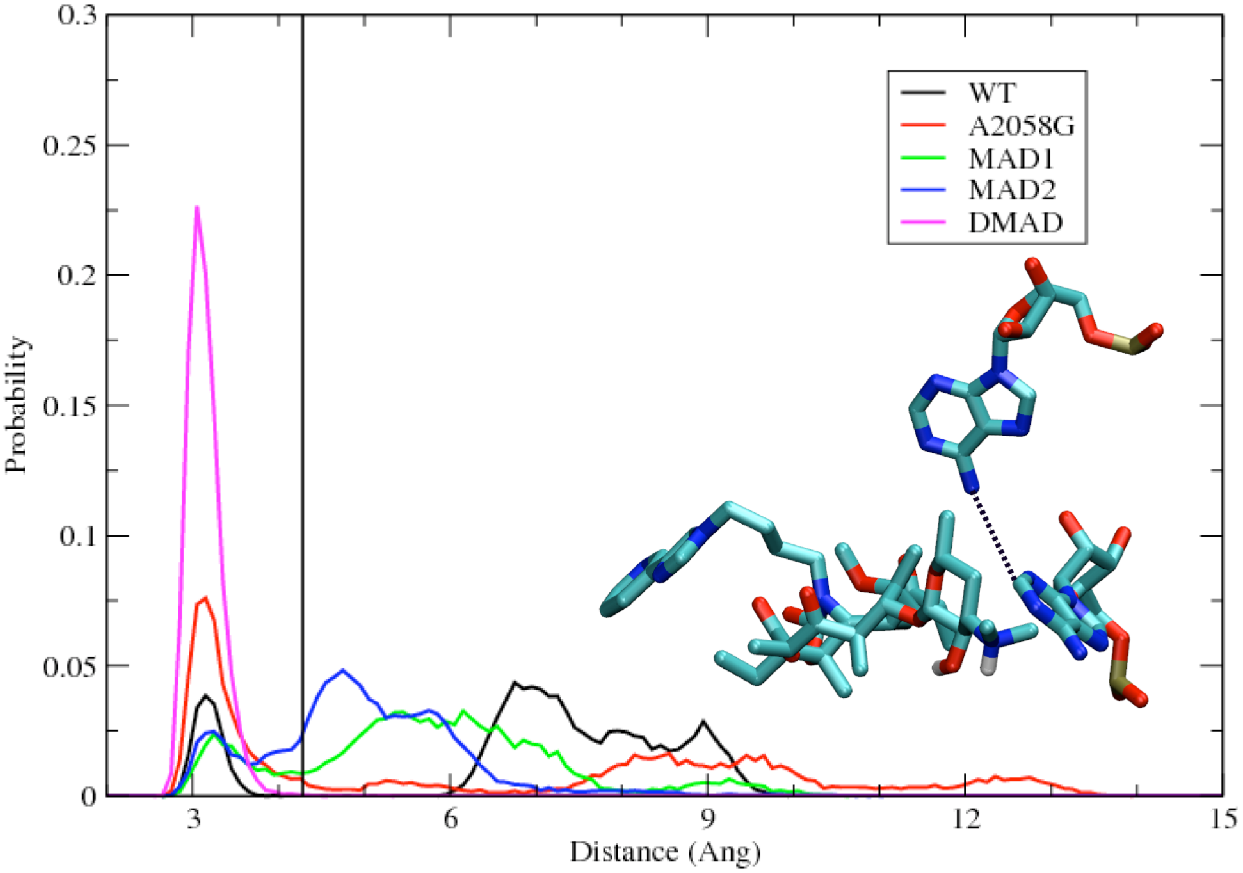

Figure S10. The probability distributions of distances for A2059 C2 to A2062 N6. WT is shown in black, A2058G in red, MAD1 in green, MAD2 in blue, and DMAD in magenta. The crystal structure distance is shown as a black vertical line.

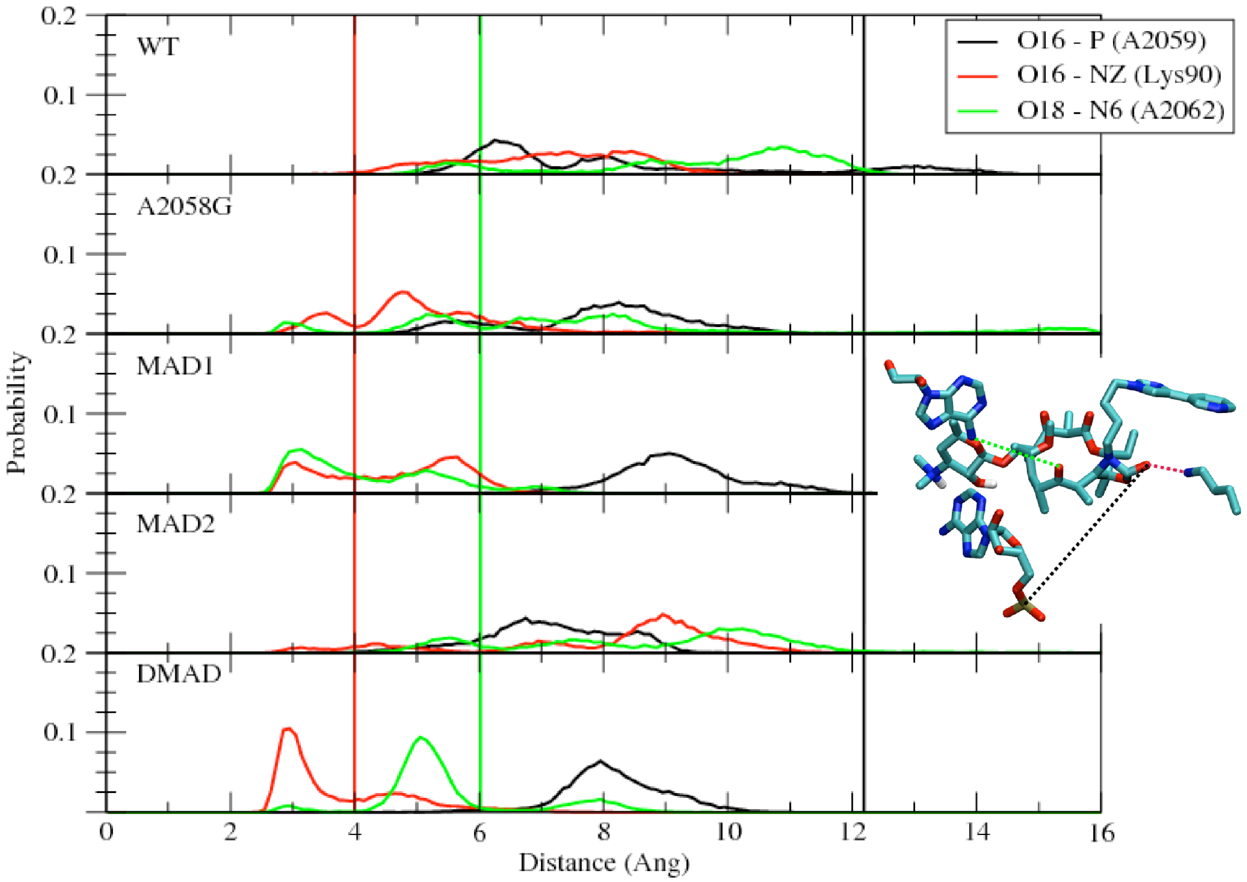

Figure S11. The probability distributions for the distances between telithromycin O16 to A2059 P (black), O16 to Lys90 NZ (red), and O18 to A2062 N6 (green). Corresponding crystal structure distances are illustrated as vertical lines using the same color scheme. The inset figure illustrates the distances and orientations of the atoms from the crystal structure. O16 refers to C16-ketone, O18 refers to C9-ketone.

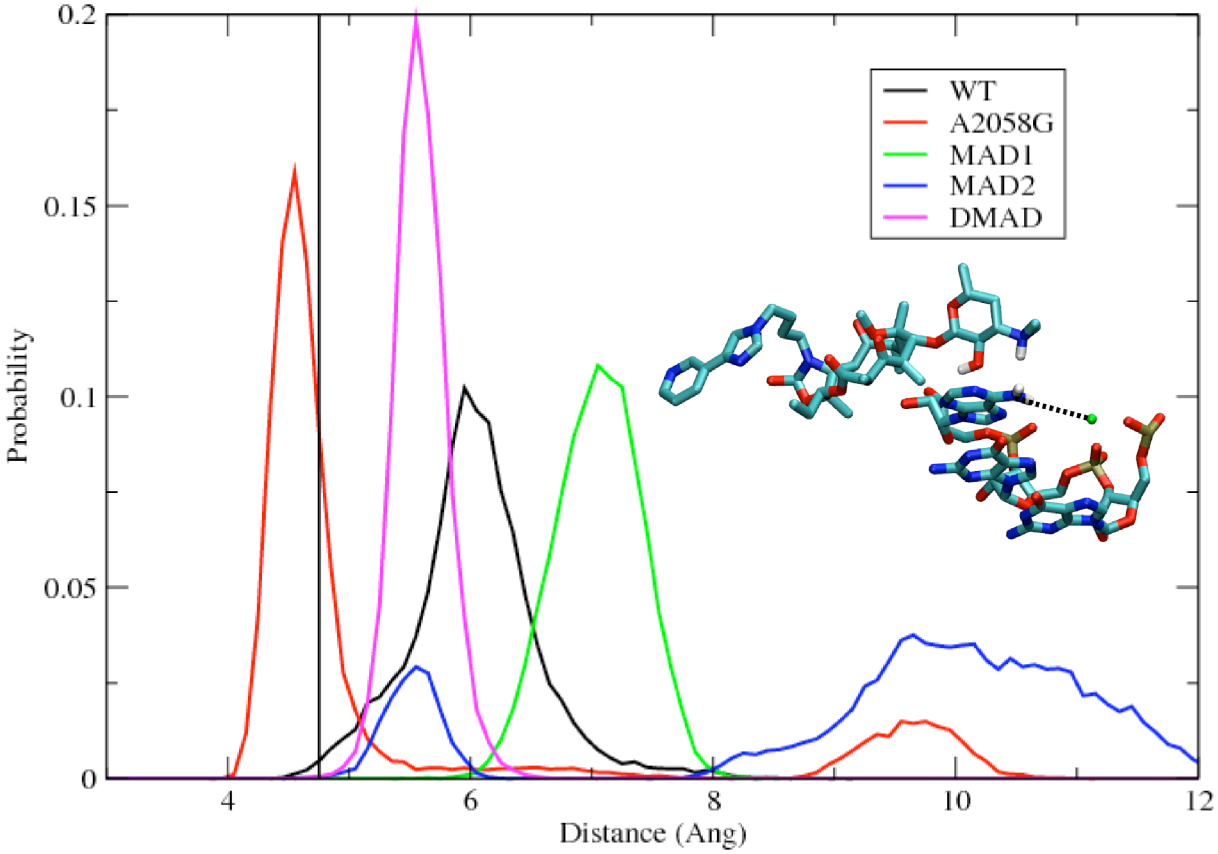

Figure S12. The probability distribution of distances between 2058 N6/O6 and a nearby Mg^{2+} ion that chelates the phosphate groups of G2056 and G2057 and two crystallographic waters. Note that Mg^{2+} and crystallographic waters were included in the simulations. Waters are excluded in the image for clarity. WT is shown in black, A2058G in red, MAD1 in green, MAD2 in blue, and DMAD in magenta. The crystal structure distance is shown as a vertical black line.

Table S1. MIC values (ug/mL) for telithromycin and erythromycin (in parentheses) against WT, A2058G mutant, and mono/dimethyl modifications. References are reported with the values. For details of measurements and species-specific information (i.e. strain, plasmids for methylation, etc.), please refer to the references.

Species	WT	A2058G	N6-monomethyl	N6,N6'-dimethyl
E. coli [22]	0.5 (0.5)		2 (128)	512 (1024)
M. smegmatis [23]	0.25 (16)	128 (>1024)		

Table S2. Interaction Energies (kcal/mol) and Distances (Å) for N6-monomethyl Adenine and Water Complexes.

Interaction geometry	ΔE (HF)*	ΔE (36NA)	ΔΔE (36NA-HF)	r (HF)	r (36NA)	Δr (36NA- HF)
H71...OHH	-1.71	-1.54	0.17	2.65	2.65	0.00
H72...OHH	0.06	-0.32	-0.38	2.77	2.71	-0.06
H73...OHH	-0.78	-0.32	0.46	2.75	2.71	-0.04
H62...OHH	-1.74	-1.51	-0.23	2.17	2.02	-0.15
N6.....HOH	-2.88	-2.95	-0.07	2.35	2.31	-0.04
**AD**<br>**RMSD**<br>**AAD**			0.08<br>0.29<br>0.26			

* HF energies are scaled by a factor of 1.16. Water interactions are shown only for a subset of atoms pertaining to the added methyl group and surrounding atoms. Included are average deviation (AD), root mean square deviation (RMSD), as well as absolute average deviation (AAD). Atom numbering is as follows (**Figure S1**): H71, H72, and H73 correspond to the exocyclic methyl hydrogens, H62 is the exocyclic amine hydrogen, and N6 is the exocyclic amine nitrogen.

Table S3. Interaction Energies (kcal/mol) and Distances (Å) for N6,N6'-dimethyl Adenine and Water Complexes.

**Interaction geometry**	**ΔE (HF)***	**ΔE (36NA)**	**ΔΔE (36NA- HF)**	**r (HF)**	**r (36NA)**	**Δr (36NA- HF)**
H71...OHH	-1.59	-1.13	0.46	2.67	2.70	0.03
H72...OHH	0.64	0.31	-0.33	3.28	2.85	-0.43
H73...OHH	-0.74	-0.13	0.61	2.79	2.76	-0.03
H74...OHH	-1.37	-1.29	0.08	2.69	2.69	0.00
H75...OHH	-0.94	-0.16	0.78	2.78	2.74	-0.04
H76...OHH	-1.51	0.64	2.15	5.68	2.82	-2.86
N6.....HOH	-2.28	-2.80	-0.52	2.33	2.19	-0.14
**AD**<br>**RMSD**<br>**AAD**			0.46<br>0.82<br>0.71			

* HF energies are scaled by a factor of 1.16. Water interactions are shown only for a subset of atoms pertaining to the added methyl group and surrounding atoms. Included are average deviation (AD), root mean square deviation (RMSD), as well as absolute average deviation (AAD. Atom numbering is as follows : H71, H72, and H73 correspond to the exocyclic methyl hydrogens for the methyl group C7A; H74, H75, and H76 correspond to the exocyclic methyl hydrogens for the methyl group C7B; and N6 is the exocyclic amine nitrogen. See Figure S1.

Table S4. N6-monomethyl Adenine QM and MM Vibrational Spectra for the modes involving the methylated amino group.

	**MP2/6-31G***		**CGenFF**	
Assignment	*Frequency*	*%*	*Frequency*	*%*
sN6-H	3437.7	100	3503.6	100
sN6-C7A	994.5<br>1127.6<br>1174.3	47<br>19<br>16	955.2<br>1087.9	16<br>37
sC7A-H	2925.8<br>3011.2<br>3050.4	100<br>100<br>99	2978.5<br>3022.2<br>3022.9	99<br>100<br>100
dC6N6C7A	170.9<br>326.7	20<br>34	184.5<br>334.0	32<br>37
dN6H	1447.8	22	1385.3<br>1507.2	18<br>23
wN6H62	82.2<br>428.6	44<br>23	417.3<br>471.8	52<br>19
dsC7AH	1411.6	67	1449.9	98
daC7AH	1479.7<br>1502.6	57<br>25	1406.3	84
daC7AH'	1454.1	86	1413.6	84
rC7AH	1072.3<br>1174.3	25<br>33	1043.6<br>1098.4	32<br>27
rC7AH'	1116.6	78	1065.5<br>1413.6	81<br>15
torN6-C6	82.2<br>428.6	25<br>53	129.2<br>280.4<br>332.5	26<br>39<br>27
torN6-C7A	96.2	73	96.0	99

* MP2/6-31G* values are scaled by 0.943[11]

Table S5. N6,N6'-dimethyl Adenine QM and MM Vibrational Spectra for the modes involving the methylated amino group.

	**MP2/6-31G***		**CGenFF**	
Assignment	*Frequency*	*%*	*Frequency*	*%*
sN6-C7A(B)	830.3<br>914.0<br>1049.7<br>1245.2	15<br>38<br>23<br>46	913.6<br>1289.8<br>1335.0	34<br>53<br>19
sC7B-H	2899.3<br>2985.5<br>3071.2	98<br>100<br>99	2977.7<br>2978.4<br>3020.0<br>3020.8<br>3023.4	79<br>20<br>48<br>93<br>54
dN6C7A	368.7	50	331.0<br>349.3<br>488.1	34<br>18<br>20
dN6C6	356.6	50	229.4<br>334.4<br>359.0	17<br>19<br>34
wN6C6	91.5<br>203.0<br>224.3	28<br>24<br>20	208.0<br>378.9	21<br>22
dsC7BH	1423.7	79	1462.0<br>1483.9	44<br>28
daC7BH	1474.4<br>1477.3	56<br>20	1411.5	62
daC7BH'	1462.6<br>1477.3	17<br>51	1418.5<br>1425.2	56<br>18
rC7BH	1090.8<br>1133.2	20<br>26	1041.9	47
rC7BH'	1090.8<br>1133.2	21<br>23	1022.1<br>1094.6	47<br>30
torN6-C6	67.4	80	74.9	78
torN6-C7A(B)	124.5<br>148.1<br>231.2	77<br>60<br>18	122.6<br>148.9	75<br>94

* MP2/6-31G* values are scaled by 0.943. [11]

Table S6. QM versus MM relative energies for MAD1 versus MAD2.

Energy Type (kcal/mol)*	*MAD1*	*MAD2*
**QM**	0.00	1.25
**MM**	0.00	1.30

* Energy values shown are relative to MAD1.

Table S7. The number of GCMC waters within 10 Å of telithromycin averaged over the last 25 ns of GCMC/MD for varying lengths of MD. Water molecules with oxygen atoms within 10 Å of the center are considered.

System	# waters
Original (MD=20 ps)	52
New MD=20 ps	42
MD=50 ps	42
MD=100 ps	43
MD=500 ps	42
MD=1 ns	42

REFERENCES

1. Vanommeslaeghe K, Hatcher E, Acharya C, Kundu S, Zhong S, et al. (2010) CHARMM general force field: A force field for drug-like molecules compatible with the CHARMM all-atom additive biological force fields. J Comput Chem 31: 671-690.
2. Vanommeslaeghe K, MacKerell AD, Jr. (2012) Automation of the CHARMM General Force Field (CGenFF) I: bond perception and atom typing. J Chem Inf Model 52: 3144-3154.
3. Vanommeslaeghe K, Raman EP, MacKerell AD, Jr. (2012) Automation of the CHARMM General Force Field (CGenFF) II: assignment of bonded parameters and partial atomic charges. J Chem Inf Model 52: 3155-3168.
4. MOE MOE (2012) Chemical Computing Group. 1010 Sherbooke St West, Suite #910, Montreal, QC, Canada, H3A, 2R7, 2012.
5. Denning EJ, Priyakumar UD, Nilsson L, Mackerell AD, Jr. (2012) Impact of 2'-hydroxyl sampling on the conformational properties of RNA: update of the CHARMM all-atom additive force field for RNA. J Comput Chem 32: 1929-1943.
6. Foloppe N, MacKerell AD, Jr. (2000) All-atom empirical force field for nucleic acids: 1) Parameter optimization based on small molecule and condensed phase macromolecular target data. J Comp Chem 21: 86-104.
7. Hart K, Foloppe N, Baker CM, Denning EJ, Nilsson L, et al. (2012) Optimization of the CHARMM additive force field for DNA: Improved treatment of the BI/BII conformational equilibrium. J Chem Theory Comput 8: 348-362.
8. MacKerell Jr. AD, Banavali N, Foloppe N (2000) Development and current status of the CHARMM force field for nucleic acids. Biopolymers 56: 257-265.
9. Brooks BR, Brooks III CL, Mackerell AD, Jr., Nilsson L, Petrella RJ, et al. (2009) CHARMM: the biomolecular simulation program. J Comput Chem 30: 1545-1614.
10. Frisch MJ, Trucks GW, Schlegel HB, Scuseria GE, Robb MA, et al. (2004) Guassian03. Wallingford, CT: Gaussian, Inc.
11. Scott AP, Radom L (1996) Harmonic Vibrational Frequencies: An Evaluation of Hartree-Fock, Moller-Plesset, Quadratic Configuration Interaction, Density Functional Theory, and Semiempirical Scale Factors. J Phys Chem 100: 16502-16513.
12. Sanbonmatsu KY, Joseph S, Tung CS (2005) Simulating movement of tRNA into the ribosome during decoding. Proc Natl Acad Sci U S A 102: 15854-15859.
13. Sanbonmatsu KY, Tung CS (2007) High performance computing in biology: multimillion atom simulations of nanoscale systems. J Struct Biol 157: 470-480.
14. Brooks III CL, Brunger A, Karplus M (1985) Active site dynamics in protein molecules: a stochastic boundary molecular-dynamics approach. Biopolymers 24: 843-865.
15. Brooks III CL, Karplus M (1983) Deformably stochastic boundaries in molecular dynamics. J Chem Phys 79: 6312-6325.
16. Brooks III CL, Karplus M (1989) Solvent effects on protein motion and protein effects on solvent motion. Dynamics of the active site region of lysozyme. J Mol Biol 208: 159-181.
17. Brunger A, Brooks III CL, Karplus M (1984) Stochastic boundary conditions for molecular dynamics simulations of ST2 water. Chem Phys Lett 105: 495-500.
18. Im W, Berneche S, Roux B (2001) Generalized solvent boundary potential for computer simulations. J Chem Phys 114: 2924-2937.
19. Hu J, Ma A, Dinner AR (2006) Monte Carlo simulations of biomolecules: The MC module in CHARMM. J Comput Chem 27: 203-216.

20. Woo HJ, Dinner AR, Roux B (2004) Grand canonical Monte Carlo simulations of water in protein environments. J Chem Phys 121: 6392-6400.
21. Metropolis N, Rosenbluth AW, Rosenbluth MN, H. TA (1953) Equation of state calculations by fast computing machines. J Chem Phys 21: 1087-1092.
22. Liu M, Douthwaite S (2002) Activity of the ketolide telithromycin is refractory to Erm monomethylation of bacterial rRNA. Antimicrob Agents Chemother 46: 1629-1633.
23. Pfister P, Jenni S, Poehlsgaard J, Thomas A, Douthwaite S, et al. (2004) The structural basis of macrolide-ribosome binding assessed using mutagenesis of 23S rRNA positions 2058 and 2059. J Mol Biol 342: 1569-1581.
